# Preterm Brain Injury and Neurodevelopmental Outcomes: A Meta-analysis

**DOI:** 10.1542/peds.2022-057442

**Published:** 2022-11-04

**Authors:** Philippa Rees, Caitriona Callan, Karan R. Chadda, Meriel Vaal, James Diviney, Shahad Sabti, Fergus Harnden, Julian Gardiner, Cheryl Battersby, Chris Gale, Alastair Sutcliffe

**Affiliations:** a Population Policy and Practice, Great Ormond Street UCL Institute of Child Health, London, United Kingdon; b Nuffield Department of Primary Care Health Sciences, University of Oxford, Oxford, United Kingdom; c Department of Paediatrics, Cambridge University Hospitals NHS Foundation Trust, Cambridge, United Kingdom; d Paediatric ICU, Great Ormond Street Hospital, London, United Kingdom; e Kings College London, London, United Kingdom; f Chelsea and Westminster Hospital NHS Foundation Trust, London, United Kingdom; g

## Abstract

**CONTEXT:**

Preterm brain injuries are common; neurodevelopmental outcomes following contemporary neonatal care are continually evolving.

**OBJECTIVE:**

To systematically review and meta-analyze neurodevelopmental outcomes among preterm infants after intraventricular hemorrhage (IVH) and white matter injury (WMI).

**DATA SOURCES:**

Published and grey literature were searched across 10 databases between 2000 and 2021.

**STUDY SELECTION:**

Observational studies reporting 3-year neurodevelopmental outcomes for preterm infants with IVH or WMI compared with preterm infants without injury.

**DATA EXTRACTION:**

Study characteristics, population characteristics, and outcome data were extracted.

**RESULTS:**

Thirty eight studies were included. There was an increased adjusted risk of moderate-severe neurodevelopmental impairment after IVH grade 1 to 2 (adjusted odds ratio 1.35 [95% confidence interval 1.05–1.75]) and IVH grade 3 to 4 (adjusted odds ratio 4.26 [3.25–5.59]). Children with IVH grade 1 to 2 had higher risks of cerebral palsy (odds ratio [OR] 1.76 [1.39–2.24]), cognitive (OR 1.79 [1.09–2.95]), hearing (OR 1.83 [1.03–3.24]), and visual impairment (OR 1.77 [1.08–2.9]). Children with IVH grade 3 to 4 had markedly higher risks of cerebral palsy (OR 4.98 [4.13–6.00]), motor (OR 2.7 [1.52–4.8]), cognitive (OR 2.3 [1.67–3.15]), hearing (OR 2.44 [1.42–4.2]), and visual impairment (OR 5.42 [2.77–10.58]). Children with WMI had much higher risks of cerebral palsy (OR 14.91 [7.3–30.46]), motor (OR 5.3 [3–9.36]), and cognitive impairment (OR 3.48 [2.18–5.53]).

**LIMITATIONS:**

Heterogeneity of outcome data.

**CONCLUSIONS:**

Mild IVH, severe IVH, and WMI are associated with adverse neurodevelopmental outcomes. Utilization of core outcome sets and availability of open-access study data would improve our understanding of the nuances of these outcomes.

Prematurity is the leading global cause of childhood morbidity and mortality.^1,2^ Internationally, 15 million infants are born preterm every year and this figure has remained static in high-income countries since the turn of the millenium.^1,3,4^ In the last 3 decades there have been substantial changes to routine neonatal care with the widespread uptake of treatments such as antenatal steroids, careful thermoregulation, use of novel approaches, such as postnatal exogenous surfactant administration, and use of less invasive ventilation strategies. These improvements have resulted in considerable survival gains for preterm infants, and many have also been neuroprotective, and as such, have reduced the rate and severity of preterm brain injuries.^4–9^ Therefore, the relationship between neurodevelopmental impairment and brain injuries of prematurity may be different to that described previously.

Neonatal trials and observational studies typically employ composite primary outcomes (using a combination of death and disability) at 2-years of age. There are widely acknowledged issues with the use of composite outcomes, including their lack of pragmatic utility for clinicians, that they are less meaningful to parents, and that they can both mask or inflate effect sizes.^10–12^ Additionally, neonatal studies with composite primary outcomes are typically not adequately powered to explore the risk of specific neurodevelopmental sequelae after preterm brain injury, which has been repeatedly highlighted as a priority question from parents.^12,13^ A meta-analysis exploring neurodevelopmental impairment after intraventricular hemorrhage (IVH) in 2014 was only able to explore a handful of neurodevelopmental outcomes because of such issues. The included studies, even on pooling in meta-analyses, were inadequately powered to explore key outcomes, such as hearing and visual impairment, and few studies provided adjusted effect estimates.^14^ As such, and in view of the evolution of neonatal care, we anticipated that an updated overview of the evidence would prove useful and that the additional power afforded by more recent population-based studies would enable more detailed exploration of the risk of specific neurodevelopmental sequelae after preterm brain injury. Therefore, we undertook a systematic review to explore neurodevelopmental outcomes up to 3 years of age after preterm brain injuries including IVH and white matter injury (WMI).

## Methods

### Study Selection

This review followed an a priori registered protocol (CRD 42021278572). It is reported in-line with the PRISMA and MOOSE statements. Observational studies published between 2000 and 2021 examining neurodevelopmental outcomes up to 3 years of age after preterm brain injury were included. Studies were required to have a non–brain injured preterm comparator group for inclusion. Preterm brain injuries included intracranial hemorrhage, such as IVH of any grade, and WMI, such as non-cystic and cystic periventricular leukomalacia (PVL), among neonates born at less than 37 weeks’ gestation. The primary review outcome was any neurodevelopmental impairment; secondary outcomes included: cognitive, motor, speech and language, behavioral and neuropsychological, visual, and hearing impairment (Table 1).

**TABLE 1:** Inclusion and Exclusion Criteria

Inclusion Criteria	Exclusion Criteria
Peer-reviewed observational studies	Noncomparative studies; opinions; commentaries; reviews; case-reports; animal studies.
Studies in all languages	Studies where the population includes adults and children and the data for children cannot be extracted.
Studies published after 2000	Studies where comparable outcome data from those with and without preterm brain injury cannot be extracted.
Preterm children born at <37 wk’ gestation with a diagnosis of intracranial hemorrhage or white matter injury during the neonatal period as defined by authors (including on imaging review [cranial ultrasound or MRI] by neonatologists, radiologists or sonographers; or on clinical record review).^71,72^	Studies reporting outcomes for children diagnosed with preterm brain injury beyond the neonatal period.
Studies focused on neurodevelopmental outcomes of children up to 3 y of age including:	Studies not reporting quantitative neurodevelopmental, health or educational outcomes.
Primary outcome(s): neurodevelopmental impairment, as defined by authors (including direct testing, clinical record review, and parental interview or survey).	
Secondary outcome(s): (1) Any cognitive impairment, as defined by authors (direct testing). (2) Mild cognitive impairment (developmental quotient or IQ from 2 to 1 standard deviations below the mean). (3) Moderate-severe cognitive impairment (developmental quotient or IQ more than 2 standard deviations below the mean). (4) Epilepsy, as defined by authors (including medical history taking, clinical record review and parental interview or survey). (5) Emotional-behavioral difficulty, as defined by authors (including direct testing, clinical record review, and parental interview or survey). (6) Speech and language impairment, as defined by authors (on direct testing). (7) Visual impairment, as defined by authors (including direct testing, clinical record review, and parental interview or survey). (8) Hearing impairment, as defined by authors (including direct testing, clinical record review, and parental interview or survey). (9) Motor impairment, as defined by authors (including direct testing, clinical record review, and parental interview or survey). (10) Visual-motor impairment, as defined by authors (on direct testing).	

### Search Strategy

A comprehensive search strategy was developed in Medline Ovid consisting of 99 key terms and Mesh headings, which was adapted for other databases (Supplemental Fig 13). The published and gray literature were searched across 10 databases from January 1, 2000 to September 1, 2021 (Supplemental Fig 14). Searches were augmented with snowballing techniques, such as handsearching the reference lists of full-text articles.

### Study Screening and Risk of Bias

Each record identified underwent screening by 2 reviewers (P.R., C.C., M.V., J.D., S.S.) independently. The full text articles of all potentially relevant studies were retrieved and reviewed in detail by 2 trained reviewers, independently. This review included a risk of bias assessment using the Newcastle Ottawa Tool for cohort or case-control studies.^15^ Studies were assessed against 3 key domains: population selection, the comparability of the “exposed” brain injured and “comparator” non-brain injured groups; and outcome assessment (for cohort studies) or exposure assessment (for case control studies). For each domain, studies were classified as poor, fair, or good, and given an overall classification of high, moderate, or low risk of bias. Disagreements were resolved through group discussion.

### Data Extraction and Synthesis

A purpose-built Microsoft excel spreadsheet was created to extract data from included studies. Studies were stratified by brain injury type, age of outcome assessment, and outcome type. Specific outcomes for each brain injury type were described in a narrative synthesis. Where suitable data were available and studies demonstrated reasonable clinical and contextual homogeneity (in terms of population, injury type, outcome type, definitions, and assessment) data were pooled in random-effects meta-analyses using RevMan 5.4.^16^ Dichotomous data were pooled using the Mantel-Haenszel method. Where studies only presented analysis data (such as risk estimates), their data were pooled with dichotomous data from other studies using the generic inverse variance method.^16^ Statistical heterogeneity was assessed using the *I^2^* statistic. Where meta-analyses demonstrated substantial heterogeneity (>85%), sensitivity analyses were undertaken to further explore the underlying explanation for the heterogeneity based on risk of bias assessments, outcome assessment tools, and year of cohort.

## Results

### Overview

**FIGURE 1:**
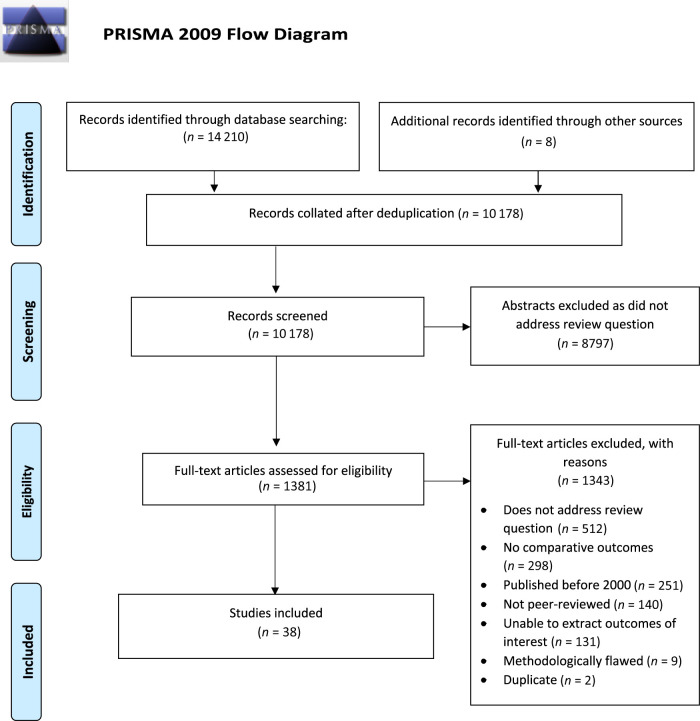
PRISMA flow diagram of included and excluded studies.

Of the 14 210 records identified, 10 178 were screened, 1381 full text articles were reviewed, and 38 studies included (Fig 1).^17–54^ Most (*n* = 35) included studies were retrospective or prospective cohort studies; 3 were case control studies. Studies were included from the United States (*n* = 17), Canada (*n* = 5), Taiwan (*n* = 4), Australia (*n* = 2) and many other countries (Supplemental Fig 15). Most studies were assessed as having a low risk of bias (*n* = 33), however 5 were deemed to have a moderate risk of bias (Supplemental Fig 16). Studies used 38 different types of outcome assessment tools and assessed outcomes at a variety of different time-points between 6 months to 3 years of age.

### IVH

Neurodevelopmental outcomes after IVH were explored by 34 included studies. Only 16 studies specified how IVH was confirmed: by radiologists (*n* = 8); neonatologists (*n* = 2); both neonatologists and radiologists (*n* = 3); or central reviewers and sonographers (*n* = 3). Five studies employed double-blinded image review. Most (*n* = 15) used the Papile classification.^17,19,22,25,31, 35,36,38,41,42,44–48^ No studies presented outcomes by laterality of IVH. In most studies, infants were born between 23 to 34 weeks’ gestation or had a birth weight of less than 1500g, and were born between 1985 and 2018, with most born after 2000 (*n* = 25).

### Outcomes After IVH Grade 1 to 2

Of the 38 included studies, 15 explored outcomes after IVH grade 1 to 2, 11 of these presented only combined outcome data for IVH grade 1 and 2. Meta-analyses comparing outcomes following grade 1 IVH alone were therefore not possible.^20,22,25,29,42,44,46,47,50,52,54^

#### Neurodevelopmental Impairment

**FIGURE 2:**
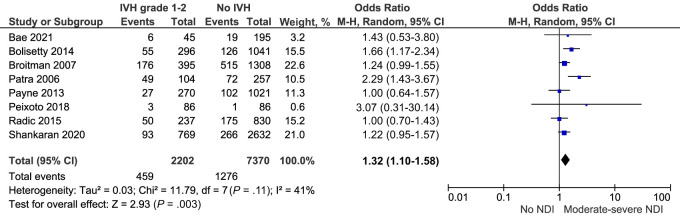
Forest plot of the crude risk of neurodevelopmental impairment after IVH grade 1 to 2. NDI, neurodevelopmental impairment.

**FIGURE 3:**
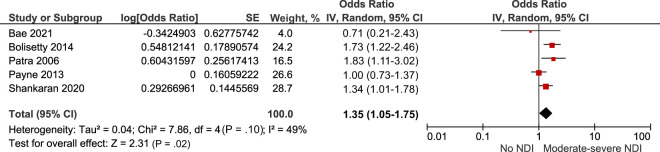
Forest plot of the adjusted risk of neurodevelopmental impairment after IVH grade 1 to 2. NDI, neurodevelopmental impairment.

Nine studies explored moderate to severe neurodevelopmental impairment at 18 to 36 months of age after IVH grade 1 to 2.^17,22,25,27,44,46–48,50^ This composite outcome included cerebral palsy, visual, hearing, or cognitive impairment (defined as 1 or 2 standard deviations below the mean on either the Bayley Scale of Infant Development [BSID II] Mental Development Index [MDI], the Bayley Scales of Infant and Toddler Development Edition 3 [Bayley-III], or the Griffiths Scale of Child Development). Of these 9 studies, 8 were deemed sufficiently comparable for meta-analysis.^22,25,27,44,46–48,50^ The meta-analysis included 2202 preterm infants with IVH grade 1 to 2 and 7370 preterm infants without IVH. Compared with preterm infants without IVH, the combined crude risk of neurodevelopmental impairment after IVH grade 1 to 2 was higher, odds ratio (OR) 1.32 95% confidence interval (CI) (1.1–1.58) *I^2^ =* 41% (Fig 2; Table 2). This remained similar on sensitivity analyses exploring the impact of outcome assessment tools (Supplemental Fig 17). Additionally, the pooled adjusted risk of neurodevelopmental impairment was higher, adjusted odds ratio (aOR) 1.35 95% CI (1.05–1.75) *I^2^* = 49% (Fig 3). Studies included in the adjusted meta-analysis accounted for several covariates, including gestation, sex, race, maternal education, and bronchopulmonary dysplasia.

**TABLE 2:** Pooled Risks of Adverse Neurodevelopmental Outcomes After Preterm Brain Injury

	IVH Grade 1–2	IVH Grade 3–4	Cystic PVL
Moderate-severe neurodevelopmental impairment	OR 1.32 (1.1–1.58) *I^2^ =* 41%, 8 studies; 9572 infants, aOR 1.35 (1.05–1.75) *I^2^* = 49%, 5 studies	OR 3.27 (2.44–4.39) *I^2^* = 81%, 7 studies, aOR 4.26 (3.25–5.59) *I^2^* = 34%, 4 studies	OR 3.63 (2.49–5.31) *I^2^* = 0%, 3 studies, aOR 2.38 (0.73–7.7) *I^2^* = 94%, 3 studies
Motor	BSID II PDI < 70, OR 1.72(0.96–3.1) *I^2^* = 82%, 3 studies; 2483 infants	BSID II PDI < 70, OR 2.7 (1.52–4.8) *I^2^* = 73%, 2 studies	BSID II PDI < 70, OR 5.3 (3–9.36) *I^2^* = 28%, 3 studies
Cerebral palsy	OR 1.76 (1.39–2.24) *I^2^* = 52%, 10 studies; 11 018 infants	OR 4.98 (4.13–6.00) *I^2^* =37%, 7 studies, moderate to severe cerebral palsy, OR 2.39 (1.49–3.85) I^2^ = 0%, 2 studies	OR 14.91 (7.3–30.46) *I^2^* = 87%, 5 studies
Cognitive impairment	BSID II MDI < 70, OR 1.79 CI (1.09–2.95) *I^2^ =* 80%, 4 studies; 3646 infants	BSID II MDI < 70, OR 2.83 (1.54–5.2) *I^2^* = 78%, 3 studies, Bayley-III scores <85, OR 2.3 (1.67–3.15) *I^2^* = 0%, 2 studies	BSID II MDI < 70, OR 3.48 95% CI (2.18–5.53) *I^2^* = 0%, 3 studies
Hearing impairment	OR 1.83 CI (1.03–3.24) *I^2^=*62%, 7 studies; 8273 infants	OR 2.44 (1.42–4.2) *I^2^* = 52%, 5 studies; 7224 infants	—
Visual impairment	OR 1.77 (1.08–2.9) *I^2^* = 0%, 6 studies; 7881 infants	OR 5.42 (2.77–10.58) *I^2^* = 50%, 5 studies; 7203 infants	—

BSID, Bayley Scale of Infant Development; MDI, Mental Development Index; PDI, Psychomotor Development Index; —, not applicable.

#### Motor and Cerebral Palsy Outcomes

Conflicting results on motor outcomes after IVH grade 1 to 2 were reported by 7 studies,^22,24,32,36,44,46,50^ 3 of which were sufficiently comparable for meta-analysis. The combined crude risk of a BSID II Psychomotor Development Index (PDI) score < 70 after IVH grade 1 to 2 was not significantly higher compared with infants without IVH, OR 1.72 95% CI (0.96–3.1) *I^2^* = 82% (Supplemental Fig 18). Shankaran 2020 reported that those with IVH grade 1 to 2 were equally likely to have “normal motor scores” as those without IVH after adjusting for confounders aOR 0.91, 95%CI (0.72–1.14). Payne 2013 also highlighted no increased adjusted risk of gross motor functional limitation after IVH grade 1 to 2 aOR 0.66, 95% CI (0.32–1.39). These studies could not be included in a meta-analysis because of heterogeneity in outcome selection and presentation.^46,50^

**FIGURE 4:**
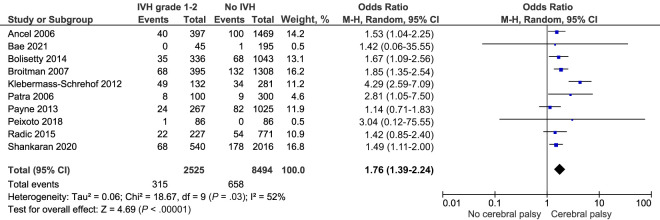
Forest plot of the crude risk of cerebral palsy after IVH grade 1 to 2.

Risk of any cerebral palsy after IVH grade 1 to 2 was reported by 10 comparable studies which included 11 018 infants.^20,22,25,27,36,44,46–48,50^ Meta-analysis finds a crude higher risk of cerebral palsy after IVH grade 1 to 2 compared to infants without IVH, OR 1.76, 95% CI (1.39–2.24) *I^2^* = 52% (Fig 4; Table 2). Sensitivity analyses exploring risk of cerebral palsy for infants born before and after 2000 did not highlight any significant differences (Supplemental Fig 19). There were insufficient data on severity of cerebral palsy and insufficient adjusted data for meta-analysis.

#### Cognitive Outcomes

Eleven included studies explored cognitive outcomes after IVH grade 1 to 2; 5 used the BSID II MDI, 4 used the Bayley-III, 1 used the Griffiths Mental Development Scales, and 1 used the Stanford Binet Intelligence Scale.^19,22,25,27,36,44,46–48,50,54^ Four studies were suitable for meta-analysis, indicating a higher crude risk of BSID II MDI <70 in infants with IVH grade 1 to 2 compared with controls, OR 1.79, 95% CI (1.09–2.95) *I^2^=*80% (Table 2; Supplemental Fig 20).^25,27,36,44^ Similar results were seen in studies not included in the meta-analysis: Peixoto 2018 reported that those with IVH grade 1 to 2 had significantly lower mean cognitive scores on the Griffiths Mental Development Scale (94.4 +/−12.7) compared with controls (98.6 +/− 9.8), but they were not more likely to have developmental quotients below 70. Payne 2013 reported that a higher risk of a BSID MDI II score <70 did not persist on adjusting for confounders aOR 1.03 (0.75–1.43).^46^ Similarly, Shankaran 2020 reported that those with IVH grade 1 to 2 had a similar risk to those without IVH of “normal” cognitive scores after adjusting for confounders (on Bayley-III): aOR 0.85, 95% CI (0.69–1.06).^50^

#### Hearing Impairment

**FIGURE 5:**
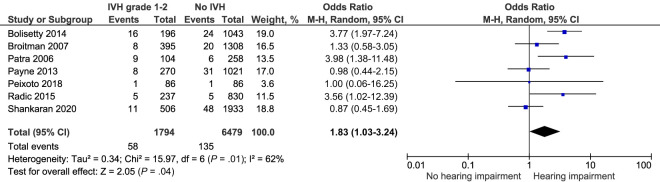
Forest plot of the crude risk of hearing impairment after IVH grade 1 to 2.

Seven included studies explored hearing impairment after IVH grade 1 to 2 among 8273 infants.^25,27,44,46–48,50^ They found a higher combined crude risk of unilateral or bilateral hearing impairment OR 1.83, 95% CI (1.03–3.24) *I^2^ =* 62% (Fig 5; Table 2). Although this outcome was rare: reported in 3.2% of infants with IVH grade 1 to 2 and 2.1% of those without IVH.

#### Visual Impairment

**FIGURE 6:**
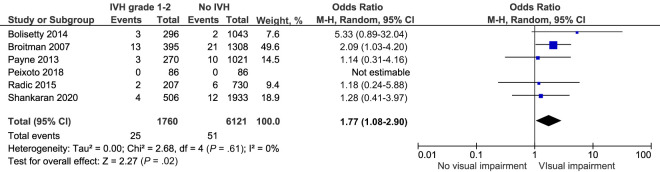
Forest plot of the crude risk of visual impairment after IVH grade 1 to 2.

The pooled crude risk of visual impairment after IVH grade 1 to 2 was significantly higher in children following IVH grade 1 to 2 compared with controls, OR 1.77, 95% CI (1.08–2.9) *I^2^* = 0% (Fig 6; Table 2), although the outcome was uncommon.^25,27,46–48,50^

### Outcomes After IVH Grade 3 to 4

Outcomes after IVH grade 3 to 4 were presented by 23 studies, most (*n* = 17) combined results for those with IVH grade 3 and 4, therefore separate meta-analyses by grade of IVH were not possible.^18,20,25,26,32–35,38,39, 41–43,46,50–52^

#### Neurodevelopmental Impairment

**FIGURE 7:**
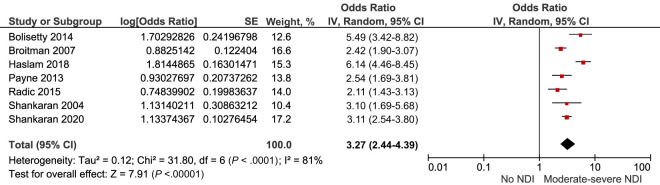
Forest plot of the crude risk of neurodevelopmental impairment after IVH grade 3 to 4. NDI, neurodevelopmental impairment.

Neurodevelopmental impairment up to 3 years after IVH grade 3 to 4 was explored by 15 studies.^17,18,23,25–27,34,35,39,41, 42,46,48,50,51^ The crude pooled risk of moderate to severe neurodevelopmental impairment was OR 3.27, 95% CI (2.44–4.39) *I^2^* = 81% (Fig 7; Table 2).

**FIGURE 8:**
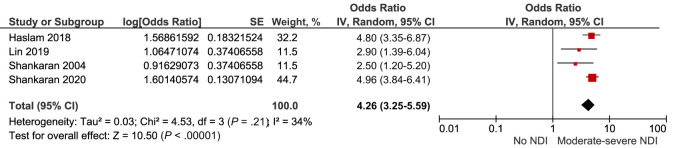
Forest plot of the adjusted risk of neurodevelopmental impairment after IVH grade 3 to 4. NDI, neurodevelopmental impairment.

Four of these studies provided adjusted measures of effect, with the risk of moderate to severe neurodevelopmental impairment persisting on pooling adjusted data (aOR 4.26, 95% CI [3.25–5.59]) *I^2^* = 34% [Fig 8; Table 2]).

#### Motor Outcomes and Cerebral Palsy

Motor outcomes after IVH grade 3 to 4 were explored by 13 studies.^18,23,24,27,32,35,36,38,41,46,48,50,51^ These studies used the BSID II PDI (*n* = 4), Bayley-III composite motor score (*n* = 6), and the Gross Motor Functional Classification System (*n* = 4). The Gross Motor Functional Classification System was used to assess the severity of functional motor impairment among those with cerebral palsy by some studies^23^; whereas others used it to assess motor function for the whole preterm study population.^32,35,46^ The combined crude risk of an abnormal BSID II PDI score (<70) across 2 comparable studies was OR 2.7, 95%CI (1.52–4.8) *I^2^* = 73% (Supplemental Fig 21; Table 2). Klebermass-Schrehof 2012 and Banihani 2019 also highlighted a significant risk of motor impairment at 2 years of age.^36,23^ De Mauro 2020, Payne 2013, and Shankaran 2020 highlighted an increased adjusted risk of major motor impairment (aOR 2.83, 95% CI [1.99–4.01]), an increased adjusted risk of gross motor functional limitations (aOR 2.51, 95% CI [1.43–4.44]), and a decreased adjusted risk of normal Bayley-III motor scores (aOR 0.37, 95% CI [0.29–0.47]) respectively.^32,46,50^

**FIGURE 9:**
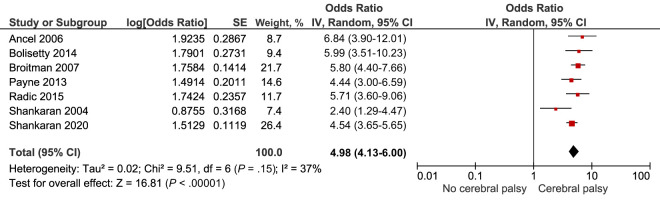
Forest plot of the crude risk of cerebral palsy after IVH grade 3 to 4.

Cerebral palsy after IVH grade 3 to 4 was explored by 11 included studies.^17,20,23,25,27,36,46,48,50–52^ Of these, 7 were suitable for meta-analysis and they highlighted a combined crude risk of OR 4.98, 95% CI (4.13–6.00) *I^2^* = 37% (Table 2; Fig 9).^20,25,27,46,48,50,51^

**FIGURE 10:**
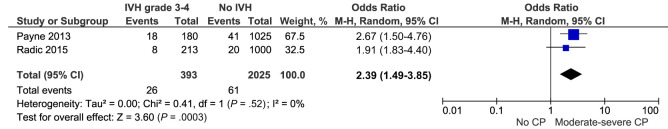
Forest plot of the crude risk of moderate to severe cerebral palsy after IVH grade 3 to 4.

Sensitivity analyses exploring risk of cerebral palsy for infants born before and after 2000 did not highlight any significant differences (Supplemental Fig 22). Two comparable studies presented data on risk of moderate to severe cerebral palsy after IVH grade 3 to 4 and highlighted a combined crude risk of OR 2.39, 95%CI (1.49–3.85) *I^2^* = 0% (Table 2; Fig 10).^46,48^

#### Cognitive Outcomes

Cognitive outcomes after IVH grade 3 to 4 were explored by 9 studies.^18,23,25,27,36,46,48,50,51^ The pooled crude risk of “abnormal” motor scores was significantly increased on BSID II (MDI < 70) OR 2.83, 95% CI (1.54–5.2) *I^2^* = 78% and Bayley-III (<85) OR 2.3, 95% CI (1.67–3.15) *I^2^* = 0% (Table 2; Supplemental Figs 23 and 24). Payne 2013 and Shankaran 2020 highlighted that this risk persisted after adjusting for confounders: the adjusted risk of a Bayley-III score <85 was aOR 1.82, 95% CI (1.26–2.64) and the adjusted risk of a normal BSID score was aOR 0.37, 95% CI (0.29–0.47) respectively.^46,50^

#### Hearing Impairment

**FIGURE 11:**
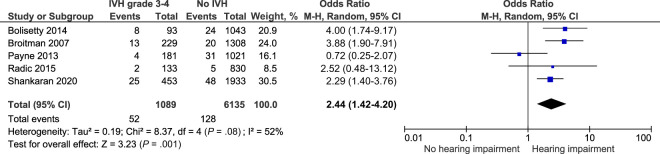
Forest plot of the crude risk of hearing impairment after IVH grade 3 to 4.

Hearing impairment after IVH grade 3 to 4 was explored by 8 included studies, 5 of these were suitable for meta-analysis.^17,25,27,43,46,48,50,55^ The combined crude risk of hearing impairment at 18 to 36 months was significantly increased OR 2.44, 95% CI (1.42–4.2) *I^2^* = 52% (Fig 11; Table 2).^25,27,46,48,50^

#### Visual Impairment

**FIGURE 12:**
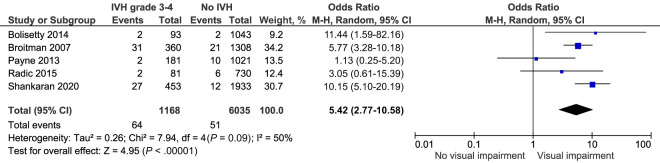
Forest plot of the crude risk of visual impairment after IVH grade 3 to 4.

Visual outcomes after IVH grade 3 to 4 were reported by 5 comparable studies that included 7203 infants. They highlighted a significantly increased crude risk of visual impairment OR 5.42, 95% CI (2.77–10.58) *I^2^* = 50% (Fig 12; Table 2).^25,27,46,48,50^

### Outcomes After WMI

#### Neurodevelopmental Impairment

Neurodevelopmental impairment up to 3 years after preterm WMI (or IVH and WMI) was explored across 12 included studies. Included infants were born at less than 34 weeks’ gestation or weighing less than 1500 g, between 1993 and 2015.^17,21,25,27,29,35,39,41,42,49,51,53^ Of these studies, 8 provided data on neurodevelopmental impairment after cystic periventricular leukomalacia (cPVL) and 4 of these were suitable for meta-analysis.^27,39,49,51^ They highlighted a significantly increased crude risk of moderate to severe neurodevelopmental impairment: OR 3.63, 95% CI (2.49–5.31) *I^2^* = 0% (Table 2; Supplemental Fig 25).^27,49,51^ This effect was attenuated on pooling studies that adjusted for key covariates, such as antenatal steroid exposure, gestation, sex, race, education, and bronchopulmonary dysplasia: aOR 2.38, 95% CI (0.73–7.7) *I^2^* = 94% (Table 2; Supplemental Fig 26).^39,49,51^ However, there was high statistical heterogeneity.

#### Motor Outcomes

Ten studies explored motor impairment (other than cerebral palsy) after WMI.^21,24,27,28,32,35,38,45,49,51^ Four studies presented motor outcomes after cPVL, 3 of which were included in a meta-analysis highlighting a higher crude risk of motor impairment (BSID II PDI <70) OR 5.3, 95% CI (3–9.36) *I^2^* = 28% (Table 2; Supplemental Fig 27).^27,32,49,51^ DeMauro 2020 also reported a higher risk of major motor abnormalities after cPVL or porencephalic cysts, which persisted after adjusting for covariates aOR 8.52, 95% CI (5.84–12.42).^32^

#### Cerebral Palsy

Nine studies reported cerebral palsy outcomes after WMI.^17,20,25,27,35,40,49,51,53^ Of these, 5 were suitable for meta-analysis and highlighted a considerably higher crude risk of cerebral palsy after cPVL OR 14.91, 95%CI (7.3–30.46) *I^2^* = 87% (Table 2, Supplemental Fig 28). Although there was considerable statistical heterogeneity, studies consistently reported an increased risk of cerebral palsy.

#### Cognitive Outcomes

Ten included studies explored cognitive outcomes after preterm WMI.^17,21,24,25,27,28,35,38,49,51^ Three studies highlighted an increased combined crude risk of cognitive impairment (BSID II MDI score < 70) OR 3.48, 95% CI (2.18–5.53) *I^2^* = 0% (Table 2, Supplemental Fig 29).

#### Behavioral and Speech and Language Outcomes

Only 1 study explored behavioral outcomes up to 3 years after WMI, and 3 explored speech and language outcomes.^21,33,38,49^ Lean 2019 and Sarkar 2018 both reported a higher crude risk of language impairment after WMI. Lean 2019 reported a higher crude risk after IVH grade 3 to 4 or posthemorrhagic hydrocephalus or cPVL OR 2.53, which did not persist on adjusting for confounders.^38^ Sarkar reported that those with disappearing cPVL (that was no longer present at 36 weeks’ gestation) had significantly lower mean language scores and as such, also had an increased crude risk of severe language impairment (Bayley-III < 70) OR 2.57, 95% CI (1.43–4.65).^49^

#### Hearing Impairment

Hearing impairment after WMI was evaluated by 3 included studies, however only 1 presented extractable outcome data.^17,25,27^ Broitman 2007 reported a higher crude risk of hearing impairment after cPVL OR 4.11, 95% CI (1.18–14.32), however this was not significant for those with noncystic PVL OR 2.5, 95% CI (0.92–6.76).^27^

#### Visual Impairment

Three studies explored visual outcomes after WMI.^17,27,45^ Broitman 2007 reported a higher crude risk of severe visual impairment after cPVL OR 13.45, 95% CI (5.8–31.18) and after PVL OR 7.15, 95% CI (3.54–14.42).^27^ Adams-Chapman 2018 combined infants with IVH grade 3 to 4 and PVL and reported a higher adjusted risk of bilateral blindness.^17^

## Discussion

This review synthesizes the considerable evidence of higher crude and adjusted risks of moderate to severe neurodevelopmental impairment after preterm brain injury. The higher risk of adverse outcomes was also significant for individual neurodevelopmental domains, including cerebral palsy, cognitive impairment, hearing impairment, and visual impairment after preterm brain injury, and were seen following lower severity IVH grade 1 to 2. This review adds further support to previous reviews highlighting an increased crude risk of moderate to severe neurodevelopmental impairment after IVH grade 3 to 4 and new evidence that these risks are increased 4-fold and persist on adjusting for key covariates. This risk of neurodevelopmental impairment derives from two to five-fold increases in the individual risks of motor impairment, cerebral palsy, cognitive impairment, hearing impairment, and visual impairment after IVH grade 3 to 4. This review quantifies the higher crude risk of moderate to severe neurodevelopmental impairment after cPVL, although this did not persist on pooling adjusted measures of effect. We also reported markedly higher risks of motor impairment (OR 5.3, 95% CI (3–9.36), cerebral palsy OR 14.91, 95% CI (7.3–30.46) and cognitive impairment OR 3.48, 95% CI (2.18–5.53) after cPVL.

### Strengths and Limitations

This review provides a comprehensive and up-to-date overview of existing evidence of neurodevelopmental outcomes after preterm brain injuries. An extensive search strategy was employed alongside a rigorous review process. Several recent population-based studies deemed to be low risk of bias were included. This enabled the review to expand on previous reviews in this area, provide stronger evidence of the risk of certain outcomes (for example by permitting new meta-analyses using adjusted data), and present novel evidence of associations with rarer outcomes, such as visual and hearing impairment. Despite this, the review findings were limited by the heterogeneity of included studies, particularly in relation to outcome assessment, outcome definitions (of neurodevelopmental impairment for example), how results were presented, and included population (with varying gestational age for example). There was also likely heterogeneity as a result of survival bias—differences in survival between studies—and potential publication bias. Unfortunately, studies combined their populations in different ways, explored varying outcomes measured with different neurodevelopmental assessment tools at different time-points, and presented their results in different ways, which limited the potential for meta-analyses and represents considerable research inefficiency.^56^ Several included studies were also not primarily designed to address our review question (for example by focusing on prematurity rather than brain injury) or did not report results for individual developmental domains, which limited the data that could be extracted. Outcomes in relation to the laterality of injury were not reported by studies, despite evidence that outcomes differ for those with bilateral and unilateral injuries.^57,58^ Because of the inclusion of studies published after 2000, included data are not completely representative of current neonatal care, for example delayed cord clamping and antenatal magnesium sulfate were not routine at the time of some studies.^59,60^ In addition, there were limited available data on key covariates, such as childhood environmental factors, which could act as important outcome modifiers. Many of the larger studies included in this review used data from neonatal networks consisting of specific tertiary units and are therefore not necessarily representative of population level care and outcomes, limiting their generalizability. Previous studies highlight poor interrater reliability in determining low grades of IVH on cranial ultrasound: this could have potentially attenuated or inflated the strength of the associations presented between low grade IVH and adverse neurodevelopmental outcomes.^61^ Finally, it is difficult to assess individual neurodevelopmental domains in isolation, which may affect results.^62^

### Context of Current Literature

This review provides further evidence to support the findings of Mukerji 2015, who highlighted an increased risk of moderate to severe neurodevelopmental impairment, cerebral palsy, and cognitive impairment after IVH grade 1 to 2 and IVH grade 3 to 4.^14^ We included several additional studies in our crude and adjusted meta-analyses with resultantly reduced heterogeneity^22,34,39,47,48, 50,51^; we used random rather than fixed effects models as suggested for observational studies of heterogenous populations.^16^ We also provide new results, for example, we highlight that the risk of cognitive impairment after IVH grade 3 to 4 persists on adjusting for key confounders – previous reviews were unable to demonstrate this because of a lack of data. Previous studies and reviews in this area were also not powered to explore the risk of hearing or visual impairment after IVH grade 1 to 2 as presented in this review.

Gotardo 2019 also highlight an increased crude risk of cerebral palsy after IVH grade 2 to 3, PVL, and cPVL. However, their review was narrow and limited to older prospective studies (mostly including children from the presurfactant era).^63^ Our review, in-keeping with the findings of previous reviews in this area, therefore provides an updated overview of the literature.

### Implications

Although this review provides evidence that preterm brain injuries are associated with a range of adverse neurodevelopmental outcomes, several questions could not be addressed. This was largely because of issues with how studies presented results rather than a paucity of research. Adoption of the core outcomes set and use of consistent definitions in neonatology offers a potential solution to this problem, alongside improved research transparency and provision of open access to study-data.^13^ This would increase the comparability of studies internationally and enable rigorous meta-analyses to address priority questions more efficiently. We would urge future studies to provide disaggregated outcome data based on site, laterality, severity of injury, and additional concurrent injuries to enable more granular analyses that would, in turn, inform more personalized counseling of parents.^64^

The continual evolution of neonatal care has meant that the risk of adverse neurodevelopmental sequelae for infants with preterm brain injuries born today is unclear; this is partially because of the time-lag between undertaking primary research, evidence synthesis, and publication. Improved used of routine data to monitor the incidence and outcomes of brain injuries for this population in real-time could address this problem and enable concurrent monitoring of the impact of quality improvement initiatives. Linkage to other data sources would also enable exploration of the impact of environmental factors on outcomes and efficient exploration of later childhood outcomes. In this review, we were unable to explore trajectories after preterm brain injuries, ie, to determine whether these adverse neurodevelopmental outcomes persist, worsen, or even improve throughout childhood. However, this should be a priority question in future studies as 3-year outcomes are not necessarily predictive of school-aged outcomes.^65–68^

Routine follow-up of preterm infants with these brain injuries is essential to support parents, detect signs of adverse neurodevelopmental outcomes, and intervene early to optimize outcomes. A recent Cochrane review highlighted that early developmental interventions can improve cognitive and motor outcomes of preterm infants.^69^ The potential of such interventions to exploit the neuroplasticity of the newborn brain, in the context of preterm brain injury, to mitigate adverse childhood outcomes also requires further exploration.^70^

## Conclusion

This systematic review presents updated evidence of numerous adverse neurodevelopmental outcomes associated with preterm brain injuries, many of which persist on adjusting for confounders. Our findings were limited by the heterogeneity of reported outcomes and by the often limited data presented by studies. Population studies employing a core outcomes set are needed to enable international comparisons with a view to improving our understanding of changes in outcome over time, the role of confounders and effect modifiers, and the potential for early intervention to harness the neuroplasticity of the brain and ultimately improve outcomes.

## Supplementary Material

Supplemental Information

## Abbreviations

aOR: adjusted OR
Bayley III: Bayley Scales of Infant and Toddler Development edition 3
BSID: Bayley Scale of Infant Development
CI: confidence intervals
cPVL: cystic periventricular leukomalacia
IVH: intraventricular hemorrhage
MDI: Mental Developmental Index
PDI: psychomotor development index
PVL: periventricular leukomalacia
WMI: white matter injury
