# Supplementary material for: Preterm Brain Injury and Neurodevelopmental Outcomes: A Meta-analysis
Source: Pediatrics. 2022 Nov 4;150(6):e2022057442. doi: 10.1542/peds.2022-057442 (PMC9724175; doi:10.1542/peds.2022-057442)
Supplement: Supplemental Information [file PEDS_2022057442SupplementaryData.pdf]

Supplemental Information

(N.B. Search strategy designed for a broader systematic review which included this one)
1. exp CHILD/
2. exp Child, Preschool/
3. exp ADOLESCENT/
4. exp INFANT/ or exp INFANT, NEWBORN/
5. (child* or toddler* or baby or infant* or adolescent*).mp.
6. 1 or 2 or 3 or 4 or 5
7. exp Educational Status/
8. exp Child Development/
9. exp Learning Disorders/
10. exp Educational Measurement/
11. exp SCHOOLS/
12. exp Academic Performance/
13. school performance.mp.
14. exp COGNITION/
15. exp LEARNING/
16. exp SPATIAL LEARNING/
17. exp VERBAL LEARNING/
18. exp SOCIAL LEARNING/
19. exp Intelligence Tests/
20. exp INTELLIGENCE/
21. exp Intellectual Disability/
22. exp Neurodevelopmental Disorders/
23. neurodevelopm*.mp.
24. (nervous system dys* or CNS dys*).mp. [mp=title, abstract, heading word, drug trade name, original title, device manufacturer, drug manufacturer, device trade name, keyword, floating subheading word, candidate term word]
25. (nervous system abnorm* or CNS abnorm*).mp. [mp=title, abstract, heading word, drug trade name, original title, device manufacturer, drug manufacturer, device trade name, keyword, floating subheading word, candidate term word]
26. (nervous system malform* or CNS malform*).mp. [mp=title, abstract, heading word, drug trade name, original title, device manufacturer, drug manufacturer, device trade name, keyword, floating subheading word, candidate term word]
27. (nervous system dis* or CNS dis*).mp. [mp=title, abstract, heading word, drug trade name, original title, device manufacturer, drug manufacturer, device trade name, keyword, floating subheading word, candidate term word]
28. (mental health condi* or mental health dis*).mp. [mp=title, abstract, heading word, drug trade name, original title, device manufacturer, drug manufacturer, device trade name, keyword, floating subheading word, candidate term word]
29. mental health outcome.mp.
30. behaviour* abnorm*.mp.
31. cognitive impairment.mp. or exp Cognitive Dysfunction/
32. visual impairment.mp. or exp Vision Disorders/
33. visual develop*.mp.
34. (visual dis* or visual dys*).mp. [mp=title, abstract, heading word, drug trade name, original title, device manufacturer, drug manufacturer, device trade name, keyword, floating subheading word, candidate term word]
35. (nystagmus or strabismus).mp.

SUPPLEMENTAL FIGURE 13
Medline Ovid search strategy.

36. (visual acuity or refractive error*).mp.
37. hearing impairment.mp. or exp Hearing Loss/
38. exp Deafness/
39. exp DEAF-BLIND DISORDERS/
40. exp Hearing Loss, Sensorineural/
41. exp Movement Disorders/
42. exp Cerebral Palsy/
43. motor impairment.mp.
44. (seizure* or convulsi*).mp.
45. exp EPILEPSY/ or epilepsy.mp.
46. exp Executive Function/
47. visual-motor impairment.mp.
48. numeracy.mp.
49. literacy.mp. or exp LITERACY/
50. jaundice.mp.
51. exp Language Development Disorders/ or exp Child Language/ or language impairment.mp. or exp Reading/ or exp Dyslexia/ or reading impairment.mp.
52. 7 or 8 or 9 or 10 or 11 or 12 or 13 or 14 or 15 or 16 or 17 or 18 or 19 or 20 or 21 or 22 or 23 or 24 or 25 or 26 or 27 or 28 or 29 or 30 or 31 or 32 or 33 or 34 or 35 or 36 or 37 or 38 or 39 or 40 or 41 or 42 or 43 or 44 or 45 or 46 or 47 or 48
53. 49 or 50 or 51
54. 52 or 53
55. exp JAUNDICE, NEONATAL/
56. exp JAUNDICE/
57. exp Hyperbilirubinemia, Neonatal/
58. exp Hyperbilirubinemia/
59. hyperbilirubin*.mp.
60. exp Hyperbilirubinemia, Hereditary/
61. bilirubin encephalopathy.mp.
62. bilirubin-induced neuro*.mp.
63. exchange transfusion.mp.
64. exp ASPHYXIA NEONATORUM/
65. (exp ASPHYXIA/ or asphyxia.mp.) and neonat*.mp.
66. exp Hypoxia-Ischemia, Brain/ and neonat*.mp.
67. perinatal asphyxia.mp.
68. birth asphyxia.mp.
69. (hypoxic-ischemic encephalopathy or hypoxic-ischaemic encephalopathy).mp.
70. neonatal encephalopathy.mp.
71. (exp Cerebral Hemorrhage/ or exp Intracranial Hemorrhages/ or exp Brain Ischemia/ or intracranial haemorrhage.mp. or exp Subarachnoid Hemorrhage/ or exp Stroke/) and neonat*.mp.
72. perinatal stroke.mp.
73. (central nervous system infection.mp. or exp Central Nervous System Infections/) and neonat*.mp.
74. (exp Meningoencephalitis/ or meningo-encephalitis.mp.) and neonat*.mp.
75. (MENINGITIS/ or meningitis.mp.) and neonat*.mp.
76. exp MENINGITIS, VIRAL/ and neonat*.mp.
77. (meningoencephalitis and neonat*).mp.

SUPPLEMENTAL FIGURE 13
Continued.

78. (encephalitis.mp. or exp ENCEPHALITIS, VIRAL/ or exp INFECTIOUS ENCEPHALITIS/ or exp ENCEPHALITIS/) and neonat*.mp.
79. kernicterus.mp. or exp KERNICTERUS/
80. preterm white matter disease.mp.
81. (periventricular leukomalacia.mp. or exp Leukomalacia, Periventricular/) and neonat*.mp.
82. (therapeutic hypothermia.mp. or exp Hypothermia, Induced/) and neonat*.mp.
83. ((subdural haemorrhage or subdural hemorrhage) and neonat*).mp.
84. (exp Hematoma, Subdural/ or subdural haemorrhage.mp. or exp Craniocerebral Trauma/) and neonat*.mp.
85. (intraventricular haemorrhage and neonat*).mp.
86. (tentorial tear and neonat*).mp.
87. (parenchymal haemorrhage and neonat*).mp.
88. (ventriculoperitoneal shunt.mp. or exp Cerebrospinal Fluid Shunts/ or exp Ventriculoperitoneal Shunt/) and neonat*.mp.
89. ((ventricular drain or Rickham reservoir or CSF shunt) and neonat*).mp.
90. neonatal stroke.mp.
91. (cerebrovascular accident and neonat*).mp.
92. neonatal cerebral ischaemia.mp.
93. (exp Intracranial Thrombosis/ or cerebral venous thrombosis.mp.) and neonat*.mp.
94. (seizure.mp. or exp Seizures/) and neonat*.mp.
95. 55 or 56 or 57 or 58 or 59 or 60 or 61 or 62 or 63 or 64 or 65 or 66 or 67 or 68 or 69 or 70 or 71 or 72 or 73 or 74 or 75 or 76 or 77 or 78 or 79 or 80 or 81 or 82 or 83 or 84 or 85 or 86 or 87 or 88 or 89 or 90 or 91 or 92 or 93 or 94
96. exp Cohort Studies/
97. exp Retrospective Studies/
98. (cohort* or (case$ and control$)).tw.
99. exp Cross-Sectional Studies/
100. exp Randomized Controlled Trial/
101. 96 or 97 or 98 or 99 or 100
102. exp "REVIEW"/
103. exp Case Reports/
104. Animals/
105. animal stud*.mp.
106. 102 or 103 or 104 or 105
107. 6 and 52 and 95 and 101
108. 107 not 106

SUPPLEMENTAL FIGURE 13
Continued.

Cochrane Central Register of Controlled Trials
EBSCO–CINAHL (Cumulative Index to Nursing and Allied Health Literature)
Google Scholar
Ovid–EMBASE
Ovid–MEDLINE
Ovid–MEDLINE E-pub ahead of print
Ovid–MEDLINE In-Process and Other Non-Indexed Citations
PubMed
Scopus
Web of Knowledge (Science Citation Index Expanded and Conference Proceedings Citation Index Science)

SUPPLEMENTAL FIGURE 14
Databases searched.

#overlapping study data; Bayley Scale of Infant Development (BSID); Bronchopulmonary Dysplasia (BPD); Cystic Periventricular leukomalacia (cPVL); Gross Motor Function Classification System, (GMFCS); Intraventricular hemorrhage (IVH); Mental Developmental Index (MDI); Periventricular leukomalacia (PVL); Necrotizing Enterocolitis (NEC); National Institute of Child Health and Human Development (NICHD); Neonatal Intensive Care Unit (NICU); Psychomotor Development Index (PDI); Patent Ductus Arteriosus (PDA); Respiratory Distress Syndrome (RDS), Small for Gestational Age (SGA), Spontaneous Intestinal Perforation (SIP)				
	Author<br>Year<br>Country<br>Study type	Population<br>Exposures<br>Comparator<br>Ascertainment/ definition	Outcomes	Main result(s)
1	Adams-Chapman 2018[17]<br><br>USA<br><br>Retrospective cohort	**Population**<br>• Mean gestation: 25.0 ± 1.0<br>• Born 2011-2015<br><br>**Exposures (n=364)**<br>• IVH grade 3-4 ± PVL<br>• cPVL<br><br>**Comparator (n= 1740)**<br>• Unmatched<br>• Preterm infants<br>• Without IVH grade 3-4<br><br>**Ascertainment/ definition**<br>• NICHD Neonatal Research Network<br>• Papile classification	**Outcomes**<br>• Moderate to severe neurodevelopmental impairment (composite)<br>• Cognitive outcomes<br>• Cerebral palsy<br>• Blindness<br>• Hearing impairment<br><br>**Measurement/ assessment**<br>• BSID III<br>• GMFCS<br>• Standardized neurosensory exam<br><br>**Follow-up**<br>• 21 months<br>• 89% follow-up	**<u>Adjusted logistic regression for neurodevelopmental impairment after IVH grade 3-4 or cPVL</u>**<br>• Moderate-severe neurodevelopmental impairment p<0.0001<br>• BSID III cognitive <70 p<0.0001,<br>• BSID III motor <70 p<0.001<br>• Moderate to severe CP p <0.0001<br>• GMFCS of 2 or greater p<0.0001<br>• Bilateral blindness p<0.001<br>• Hearing impairment p 0.91<br>(Only p values provided, adjusted for location of birth, year, bronchopulmonary dysplasia and maternal education)<br>**Neurological exam:**<br>Grade 3 IVH (n=148):<br>• Normal 48% (n=71)<br>• Suspect 15% (n=22)<br>• Abnormal without cerebral palsy 14% (n=20)<br>• Abnormal with cerebral palsy 24% (n=35)<br>Grade 4 IVH (n=180):<br>• Normal 29% (n=52)<br>• Suspect 15% (n=27)<br>• Abnormal without cerebral palsy 11% (n=20)<br>• Abnormal with cerebral palsy 45% (n=81)<br>Severe central nervous system injury (grade 3-4 IVH or cPVL) (n=364):<br>• Normal 38% (n=137)<br>• Suspect 15% (n=54)<br>• Abnormal without cerebral palsy 12% (n=44)<br>• Abnormal with cerebral palsy 35% (n=129)<br>Cystic periventricular leukomalacia (n=116):<br>• Normal 24% (n=28)<br>• Suspect 14% (n=16)<br>• Abnormal without cerebral palsy 9% (n=10)<br>• Abnormal with cerebral palsy 53% (n=62)
2	Adant 2019[18]<br><br>Belgium<br><br>Retrospective cohort	**Population**<br>• Gestation ≤32 weeks with and without spontaneous intestinal perforation (SIP)<br>• Born 1994-2014<br><br>**Exposure**<br>• IVH grade 3-4 (n=19)<br><br>**Comparator**<br>• Matched on gender, gestation, date of birth<br>• No IVH (n=44); normal ultrasound at discharge<br><br>**Ascertainment/ definition**<br>• Clinical record review	**Outcomes**<br>• Functional disability (composite)<br>• Cognitive<br>• Motor<br>• Visual impairment<br>• Behavioral/ mental health<br>• Wellbeing<br>• Quality of life<br>• Physical health<br><br>**Measurement/ assessment**<br>• BSID II<br>• Telephone survey (parents)<br>• PedsQL<br>• IQ testing<br><br>**Follow up**<br>• 67% follow-up at 7-11 months<br>• 41% follow-up at 18-22 months<br>• 49% follow-up at 4-10 years<br>• 86% follow-up telephone survey	**<u>Outcomes of those with SIP compared to controls without SIP – by IVH subgroup</u>**<br><br>**<u>Disability</u>**<br>aOR 8.79 95%CI (1.72, 44.86)<br><br>**<u>Multiple disabilities</u>**<br>aOR 5.97 95%CI (1.61, 22.15)<br><br>**<u>Cognitive</u>**<br>**BSID II MDI score (18-20 months)**<br>aOR 0.91 95%CI (0.76, 1.08)<br><br>**<u>Motor</u>**<br>**BSID II PDI score (18-20 months)**<br>aOR 0.95 95%CI (0.75, 1.18)<br><br>**<u>Visual outcomes</u>** (wearing glasses)<br>aOR 0.474 95%CI (0.13, 1.69)<br><br>**<u>Behavioral/ mental health disorder</u>** (including attention problems, conduct problems and autism spectrum disorders)<br>aOR 1.24 95%CI (0.32, 4.8)<br><br>**<u>PedsQL low quality of life score</u>**<br>aOR 0.87 95%CI (0.77, 0.99)<br><br>**<u>PedsQL low physical health score</u>**<br>aOR 0.82 95%CI (0.66, 1.01)
3	Altendahl 2021[19]<br><br>USA<br><br>Retrospective cohort study	**Population** (n=228)<br>• Gestation ≤30 weeks, birthweight <1,500 g, or gestational age at birth >30 weeks but with an unstable clinical course<br>• Born 2011-2018<br>• Those having ROP screening<br><br>**Exposure**<br>• IVH 1-4 (n=74)<br><br>**Comparator**<br>• Unmatched<br>• No IVH (n=117)<br><br>**Ascertainment/ definition**<br>• Retrospective chart review<br>• Ultrasound reviewed by pediatric radiologist<br>• Papile classification using worst grade of IVH seen	**Outcomes**<br>• Cognitive<br>• Language<br>• Motor<br><br>**Measurement/ assessment**<br>• BSID III<br>• Visual outcomes - pediatric ophthalmologist assessment<br><br>**Follow-up**<br>• 0-12 months; 84% follow-up<br>• 12-24 months; 63% follow-up<br>• 24-36 months; 26% follow-up	**<u>Cognitive score</u>**<br>IVH aOR 7.961 95%CI (1.147–55.244)*<br><br>**<u>Language score</u>**<br>IVH aOR 1.927 95%CI (0.593–6.263)<br><br>**<u>Motor score</u>**<br>IVH aOR 4.755 95%CI (1.266–17.859)*<br><br>(Combined scores across all age groups)
4#	Ancel 2006[20]<br><br>France<br><br>Prospective cohort	**Population**<br>• Gestation 22 - 32 weeks<br>• Born 1997<br><br>**Exposure**<br>• IVH grade 1 (n=229)<br>• IVH grade 2 (n=168)<br>• IVH grade 3 (n=53)<br>• IVH grade 4 (n=10)<br>• PVL (n=165)<br>• cPVL (n=76)<br>• Ventricular dilatation (n=98)	**Outcomes**<br>• Cerebral palsy (European cerebral palsy network definition)<br><br>**Measurement/assessment**<br>• Detailed physical and neurologic examination<br><br>**Follow-up**<br>• 2 years<br>• 83% follow-up (of survivors)	**<u>Cerebral palsy</u>**<br>**IVH**<br>IVH grade 1-2 n=40; 10.1%<br>IVH grade 3-4 n=21; 33.3%<br>No IVH n=100; 6.8%<br><br>**PVL**<br>cPVL n=43; 57.1%<br>No PVL n=90; 5.3%

SUPPLEMENTAL FIGURE 15
Overview of included studies.

		**Comparator**<br>• Unmatched<br>• No IVH (n=1469)<br>• No Ventricular dilatation (n=1831)<br>• No PVL (n=1689)<br><br>**Ascertainment/ definition**<br>• EPIPAGE study<br>• Radiologists or neonatologists undertake cranial ultrasounds		
5	Bae 2018[21]<br><br>South Korea<br><br>Retrospective cohort study	**Population**<br>• Gestation <34 weeks (mean 31)<br>• Admitted 2009-2014<br><br>**Exposure**<br>• Persistent periventricular echogenicity (PVE) (n=28)<br>• > 2 weeks<br><br>**Comparator**<br>• Matched on gestation and birthweight<br>• No PVE (n=60)<br><br>**Ascertainment/ definition**<br>• Scans undertaken by the same pediatric radiologist<br>• Medical chart review	**Outcomes**<br>• Neurodevelopmental impairment (composite)<br>• Language<br>• Motor<br>• Cognitive<br><br>**Measurement/ assessment**<br>• BSID III<br><br>**Follow-up**<br>• 12 months<br>• Follow-up 43% and 86% for control and exposed group respectively	**<u>Neurodevelopmental impairment</u>**<br>PVE n=6, 25%<br>No PVE n=5, 19%<br><br>**<u>Cognitive, median score (range)</u>**<br>PVE 90 (81-100)<br>No PVE 92 (85-105) P=0.388<br><br>**<u>Motor, median score (range)</u>**<br>PVE 86 (78-95)<br>No PVE 88 (79-100) P=0.218<br><br>**<u>Language median score (range)</u>**<br>PVE 88 (78-97)<br>No PVE 89 (80-105) P=0.122
6	Bae 2021[22]<br><br>South Korea<br><br>Prospective cohort study	**Population**<br>• Birthweight <1500g<br>• Born 2015-2017<br><br>**Exposure**<br>• IVH 1-2 (n=45)<br><br>**Comparator**<br>• Unmatched<br>• No IVH (n=195)<br><br>**Ascertainment/ definition**<br>• Ultrasound or MRI by pediatric radiologist<br>• Papile classification	**Outcomes**<br>• Neurodevelopmental impairment (composite)<br>• Cognitive<br>• Motor<br>• Language<br>• Hearing impairment<br>• Visual impairment<br>• Cerebral palsy<br><br>**Measurement/ assessment**<br>• BSID III<br><br>**Follow-up**<br>• 18-24 months<br>• 76% follow-up	**<u>Neurodevelopmental impairment</u>**<br>IVH 1-2 n=6<br>No IVH n=19 p=0.43<br><br>OR 1.43 95%CI (0.53, 3.8) p=0.497<br>Adj OR 0.71 95% CI (0.21, 2.46) p=0.594<br><br>**<u>Cognitive, median (interquartile range)</u>**<br>IVH 1-2 90 (87.5-110)<br>No IVH 100 (90-105) p=0.484<br><br>**<u>Motor, median (interquartile range)</u>**<br>IVH 1-2 97 (88-100)<br>No IVH 97 (88-103) p=0.617<br><br>**<u>Motor score <85</u>**<br>IVH 1-2 n=6, 13.3%<br>No IVH n=15.7.7% p=0.38<br><br>**<u>Cerebral palsy</u>**<br>IVH 1-2 n=0<br>No IVH n=1 p=1<br><br>**<u>Language, median (interquartile range)</u>**<br>IVH 1-2 90 (79-100)<br>No IVH 97 (86-106) p=0.025
7	Banihani 2019[23]<br><br>Canada<br><br>Retrospective cohort study	**Population**<br>• Gestation <29 weeks<br>• Born 2010-2013<br>• Survivors<br><br>**Exposure**<br>• IVH grade 4 (n=20)<br><br>**Comparator**<br>• Matched on sex, gestation, month of birth<br>• No IVH or germinal matrix hemorrhage (n=40)<br><br>**Ascertainment/ definition**<br>• Images reviewed by two neonatologists	**Outcomes**<br>• Neurodevelopmental impairment (composite)<br>• Cognitive<br>• Motor<br>• Language<br>• Cerebral palsy<br><br>**Measurement/ assessment**<br>• BSID III<br>• GMFCS<br><br>**Outcomes**<br>• 18-24 months<br>• 95% follow-up (intention to treat analysis)	**<u>Neurodevelopmental impairment</u>**<br>PHVI, n=12, 60%<br>No PVHI, n=25, 62.5%<br><br>OR 0.9 95%CI (0.3, 2.7) p=1<br><br>**<u>Cognitive</u>**<br>**Cognitive score, mean (SD)**<br>PHVI 91.4 (17.9)<br>No PHVI 90.4 (11.5) P=0.82<br><br>**Score <85**<br>PHVI n=5, 29%<br>No PHVI n=8, 20%<br>OR 1.67 95%CI (0.45, 6.11) p=0.5<br><br>**<u>Motor</u>**<br>**Motor score, mean (SD)**<br>PHVI 83.1 (14.1)<br>No PHVI 90.6 (12.4) P=0.05<br><br>**Score <85**<br>PHVI n=8, 50%<br>No PHVI n=9, 22.5%<br>OR 3.44 95%CI (1.01, 11.78) P=0.06<br><br>**Cerebral palsy**<br>PHVI n=17, 85%<br>No PHVI n=3, 7.5%<br>OR 69.89 (12.76, 382.65) P< 0.0001<br><br>**<u>Language</u>**<br>**Language score, mean (SD)**<br>PHVI 87.6 (14.0)<br>No PHVI 84.1 (13.7) P=0.38<br><br>**Score <85**<br>PHVI n=7, 41.2%<br>No PHVI n=22, 55%<br>OR 0.57 95%CI (0.18, 1.81) P=0.4
8	Benavente-Fernandez 2019[24]<br><br>Canada	**Population (n=234)**<br>• Gestation 24-32 weeks<br>• Born 2006-2013<br>• Survivors	**Outcomes**<br>• Cognitive<br>• Motor<br><br>**Measurement/ assessment**	**<u>Cognitive</u>**<br>**At 18 months, cognitive score, mean (95%CI)**<br>Brain injury 97.2 (89.1, 105.3)<br>No brain injury 102.2 (96.3, 108.2)

SUPPLEMENTAL FIGURE 15
Continued.

	Prospective cohort study	**Exposure**<br>• IVH<br>• PVL<br>• Brain injury (IVH grade 3 and PVL) (n=62)<br><br>**Comparator**<br>• Unmatched<br>• No brain injury on MRI (n=124)<br><br>**Ascertainment/ definition**<br>• Medical record review<br>• MRI reviewed by blinded neuroradiologist to determine severity	• BSID III (18-36 months)<br>• Weschler primary and preschool scale of intelligence 4th edition (4.5 years)<br>• Movement ABC (4.5 years)<br><br>**Follow up**<br>• 18 months; 88% follow-up<br>• 36 months; 83% follow-up<br>• 4.5 years; 75% follow-up	**Association with cognitive score**<br>**IVH grade 3**<br>β -7.14 (-18.95 4.68) p=0.235<br>Standardized β 0.08<br><br>**Punctate white matter injury volume**<br>β -0.01 (-0.01, -0.003) p=0.001<br>Standardized β 0.25<br><br>**At 36 months cognitive score, mean (95%CI)**<br>Brain injury 94.2 (86.1, 102.4)<br>No brain injury 99.7 (93.7, 105.7)<br><br>**Association with cognitive score**<br>**IVH grade 3**<br>β -8.04 (-19.49, 3.41) p=0.168<br>Standardized β 0.11<br><br>**Punctate white matter injury volume**<br>β -0.009 (-0.01, -0.004) p=0.0001<br>Standardized β 0.11<br><br>**At 4.5 years cognitive score, mean (95%CI)**<br>Brain injury 94.2 (85.9, 102.5)<br>No brain injury 99.5 (93.5, 105.5)<br><br>**Association full scale IQ**<br>**IVH grade 3**<br>β −9.69 (−21.29, 1.90) P=0.1<br>Standardized β 023<br><br>**Punctate white matter injury volume and full scale IQ**<br>β −0.01 (−0.01, −0.003) P=0.001<br>Standardized β 0.23
9	Bolisetti 2019[26]<br><br>Australia<br><br>Retrospective cohort study	**Population (n=1514)**<br>• Gestation 23-28+6 weeks<br>• Admitted to NICU 2007-2012<br><br>**Exposure**<br>• IVH 3-4 (n=70)<br><br>**Comparator**<br>• Unmatched<br>• IVH grade 0-2 (unable to figures)<br><br>**Ascertainment/ definition**<br>• NICU data registry<br>• Worst IVH grade seen on either side on imaging or post-mortem	**Outcomes**<br>• Neurodevelopmental delay (composite)<br>• Cerebral palsy<br>• Visual impairment (bilateral blindness)<br>• Hearing impairment (bilateral requiring hearing aids or cochlear implants)<br><br>**Measurement/ assessment**<br>• BSID III<br><br>**Follow up**<br>• 2-3 years<br>• 75% follow-up	**Moderate to severe delay**<br><br>**24 weeks gestation**<br>IVH 3-4, n=63 56%<br>IVH 0-2, n= 23, 20%<br><br>**25 weeks' gestation**<br>IVH 3-4, n=70, 33%<br>IVH 0-2, n=32, 15%<br><br>**26 weeks' gestation**<br>IVH 3-4, n=62, 20%<br>IVH 0-2, n=37, 12%<br><br>**27 weeks' gestation**<br>IVH 3-4 n=94, 25%<br>IVH 0-2 n=34, 9%<br><br>**28 weeks' gestation**<br>IVH 3-4, n=192, 39%<br>IVH 0-2 n=30, 6%<br><br>**Association with moderate to severe neurodevelopmental delay**<br>**No IVH**<br>n=unknown, 8.4%<br><br>**IVH 1-2**<br>n=unknown, 15,6%<br><br>**IVH 3-4**<br>n=22, 31.4%<br>β 1.424 SE (0.288)<br>aOR 4.16 95%CI (2.36, 7.31) p <0.001
10	Bolisetty 2014[25]<br><br>Australia<br><br>Retrospective cohort	**Population**<br>• Gestation 23-28 weeks<br>• Born 1998-2004<br><br>**Exposure**<br>• Grade 1-2 IVH (n=336)<br>• Grade 3-4 IVH (n=93)<br>• PVL, porencephalic cysts or hydrocephalus at 2 weeks n=64 (all had IVH 3-4 previously)<br><br>**Comparator (n=1043)**<br>• Unmatched<br>• Preterm infants<br>• No IVH<br><br>**Ascertainment/ definition**<br>• Papile classification<br>• Reports from neonatologists or radiologists<br>• NICU network data	**Outcomes**<br>• Moderate neurosensory impairment (composite)<br>• Severe neurosensory impairment (composite)<br>• Cerebral palsy<br>• Cognitive<br>• Blindness<br>• Hearing impairment<br>• Death<br><br>**Measurement/ assessment**<br>• BSID II MDI<br>• Griffiths Mental Development Scale<br><br>**Follow-up**<br>• 24-26 months<br>• 74.8% follow-up of survivors	**Moderate to severe neurosensory impairment**<br>No IVH (n=126; 12.1%) Reference group<br>All IVH (n=114; 26.6%) OR 2.63 95% CI (1.96,3.53)***<br>IVH 1-2 (n=74; 22%) OR 2.06, 95% CI (1.48,2.86) ***<br>Isolated IVH 1-2 without PVL, porencephaly, and ventricular enlargement (n=55; 18.6%) OR 1.66 95%CI (1.17,2.35)**<br>IVH 3-4 (n=40; 40%) OR 5.49 95%CI (3.42,8.83)***<br><br>**Cerebral Palsy**<br>No IVH (n=68; 6.5%) Reference<br>All IVH (n=63; 15.1%) OR 2.48 95%CI (1.71, 3.61)***<br>IVH 1-2 (n=35; 10.4% OR 1.72 95%CI (1.11, 2.67)*<br>Isolated IVH 1-2 without PVL, porencephaly, and ventricular enlargement (n=22; 7.4%)<br>IVH 3-4 (n=28; 30.1%) OR 5.99 95% CI (3.50, 10.21)***<br><br>**Mental developmental index or general quotient ≤2 SD**<br>No IVH (n=31; 3.4%) Reference group<br>All IVH (n=37; 9.8%) OR 3.08 95% CI (1.83, 5.19)***<br>IVH 1-2 (n=23; 7.8%) OR 2.37 95% CI (1.31, 4.28)**<br>Isolated IVH 1-2 without PVL, porencephaly, and ventricular enlargement (n=15; 5.7%) OR 1.69 95% CI (0.90, 3.19)<br>IVH 3-4 (n=14; 17.5%) OR 6.00 95%CI (2.88, 12.39)***<br><br>**Bilateral blindness**<br>No IVH (n=2; 0.2%) Reference group<br>All IVH (n=5; 1.2%) OR 6.14 95% CI (1.06, 45.77)*<br>IVH 1-2 (n=3; 0.9%) OR 4.69 95% CI (0.64, 40.16)<br>Isolated IVH 1-2 without PVL, porencephaly, and ventricular enlargement (n=3; 1%) OR 5.32 95% CI (0.88, 32.04)<br>IVH 3-4(n=2; 2.2%) OR 11.44 95% CI (1.14, 114.92)*

SUPPLEMENTAL FIGURE 15
Continued.

				**Bilateral hearing loss**<br>No IVH (n=24; 2.3%) Reference group<br>All IVH (n=28; 6.5%) OR 2.96 95%CI (1.64, 5.36)***<br>IVH 1-2 (n=20; 6.0%) OR 2.69 95% CI (1.41, 5.12)**<br>Isolated IVH 1-2 without PVL, porencephaly, and ventricular enlargement (n=16; 5.4%) OR 2.42 95%CI (1.27, 4.63)**<br>IVH 3-4 (n=8; 8.6%) OR 4.0 95% CI (1.60, 9.69)<br><br>**Death**<br>• Died before cranial ultrasound (n=133)<br>• No IVH (n=176; 11%) Reference group<br>• IVH grade 1-2 (n=81; 15.7%) OR 1.5 95%CI (1.12, 2.02)**<br>• IVH grade 3-4 (n=189; 62.2%) OR 13.25 95%CI (9.92, 17.71)***<br><br>**Multivariate analysis of risk factors for moderate to severe neurosensory impairment**<br>IVH 1-2 β 0.48 (SE 0.176); adjusted OR 1.61 95% CI (1.14-2.28); p 0.006<br>IVH 3-4 β 1.339 (SE 0.257); adjusted OR 3.81 95% CI (2.30-6.30); p <0.001<br>PVL: β 2.176 (SE 0.41); adjusted OR 8.81 95% CI (3.92–19.78); p<.001<br>(Adjusted for gestation, SGA, sex, chronic lung disease, pregnancy induced hypertension, proven systemic infection, NEC, ROP grade 3-4)<br><br>**Moderate-severe neurosensory impairment in isolated grade 1-2 IVH by gestation**<br>**23-25weeks**<br>No IVH (n=35; 18%) Reference group<br>Isolated IVH 1-2 without PVL, porencephaly, and ventricular enlargement (n=25; 24.3%) adjusted OR 1.45 95%CI (0.81, 2.60)<br>**26-28 weeks**<br>No IVH (n=79; 10%)<br>Isolated IVH 1-2 without PVL, porencephaly, and ventricular enlargement (n=30; 15.5) adjusted OR 1.64 95% CI (1.04, 2.58)*<br><br>**Total (23-28 weeks)**<br>No abnormalities on cranial ultrasound (n=114; 11.6%) Reference group<br>Isolated IVH 1-2 without PVL, porencephaly, and ventricular enlargement (n=55; 18.6%) adjusted OR 1.73 95% CI (1.22, 2.46)**<br>**(Adjusted for sex, SGA, chronic lung disease, and ROP)**<br>*P 0.05, **P 0.01 ***P 0.001
11	Broitman 2007[27]<br><br>USA<br><br>Retrospective Cohort	**Population**<br>• Birthweight 401–1000 g<br>• Born 1998- 2001<br><br>**Exposure**<br>• IVH grade 1 (n=244)<br>• IVH grade 2 (n=151)<br>• IVH grade 3 (n=215)<br>• IVH grade 4 (n=145)<br>• PVL (n=134)<br>• cPVL (n=50)<br><br>**Comparison**<br>• Unmatched<br>• Normal cranial ultrasound<br><br>**Ascertainment/ definition**<br>• NICHD Neonatal Research Network<br>• Radiology reports<br>• Used the most abnormal imaging	**Outcomes**<br>• Neurodevelopmental impairment (composite)<br>• Cognitive<br>• Motor<br>• Hearing<br>• Visual impairment<br>• Cerebral palsy<br>• Functional (feeding, walking)<br><br>**Measurement/assessment**<br>• Amiel-Tison method<br>• BSID II<br>• Clinical assessment<br><br>**Follow-up**<br>• 18–22 months<br>• Follow-up was an inclusion criterion	**Neurodevelopmental impairment**<br>Normal ultrasound n=515, 39.4%<br>IVH grade 1 n=99, 40.6%<br>IVH grade 2 n=77, 51%<br>IVH grade 3 n=119, 55.4%<br>IVH grade 4 n=101, 69.7%<br>PVL n=97, 72.4%<br>cPVL n=38, 76%<br><br>**Cognitive (BSID MDI <70)**<br>Normal ultrasound n=417, 31.9%<br>IVH grade 1 n=77, 31.5%<br>IVH grade 2 n=56, 36.9%<br>IVH grade 3 n=93, 43.3%<br>IVH grade 4 n=76, 52.6%<br>PVL n=81, 60.3%<br>cPVL n=30, 60.4%<br><br>**Motor (BSID II PDI <70)**<br>Normal ultrasound n=246, 18.8%<br>IVH grade 1 n=44, 18%<br>IVH grade 2 n=34, 22.3%<br>IVH grade 3 n=79, 36.7%<br>IVH grade 4 n=80, 55.5%<br>PVL n=71, 52.8%<br>cPVL n=32, 64.6%<br><br>**Cerebral palsy**<br>Normal ultrasound n=132, 10.1%<br>IVH grade 1 n=42, 17.2%<br>IVH grade 2 n=26, 17.2%<br>IVH grade 3 n=67, 31.3%<br>IVH grade 4 n=75, 51.4%<br>PVL n=67, 50%<br>cPVL n=32, 64%<br><br>**Non-independent walking**<br>Normal ultrasound n=101, 7.7%<br>IVH grade 1 n=26, 10.7%<br>IVH grade 2 n=14, 9.3%<br>IVH grade 3 n=54, 25.1%<br>IVH grade 4 n=61, 42.4%<br>PVL n=59, 44%<br>cPVL n=25, 50%<br><br>**Blindness**<br>Normal ultrasound n=21, 1.6%<br>IVH grade 1 n=7, 2.9%<br>IVH grade 2 n=6, 4%<br>IVH grade 3 n=15, 7%<br>IVH grade 4 n=16, 11.2%<br>PVL n=14, 10.5%<br>cPVL n=9, 18%<br><br>**Deafness**<br>Normal ultrasound n=20, 1.5%<br>IVH grade 1 n=3, 1.2%<br>IVH grade 2 n=5, 3.3%<br>IVH grade 3 n=6, 2.8%<br>IVH grade 4 n=7, 4.9%<br>PVL n=5, 3.7%

SUPPLEMENTAL FIGURE 15
Continued.

				cPVL n=3, 6.3%<br><br>**<u>Non-independent feeding</u>**<br>Normal ultrasound n=167, 12.8%<br>IVH grade 1 n=34, 13.9%<br>IVH grade 2 n=21, 13.9%<br>IVH grade 3 n=50, 23.4%<br>IVH grade 4 n=41, 28.5%<br>PVL n=39, 29.1%<br>cPVL n=16, 32%
12	Chen 2004[28]<br><br>Taiwan<br><br>Retrospective cohort	**Population**<br>• Gestation 23-25 weeks<br>• Birthweight 580-1500g<br>• Born 1996-2000<br><br>**Exposure (n=54)** (DeVries)<br>• Periventricular echogenicity (PVE) <2 weeks (n=27)<br>• Periventricular echogenicity persisting>2 weeks (n=27)<br><br>**Comparator (n=60)**<br>• Matched on gestation, birthweight and sex<br>• Normal cranial ultrasound<br><br>**Ascertainment/ definition**<br>• Medical record review	**Outcomes**<br>• Cognitive<br>• Motor<br><br>**Measurement/ assessment**<br>• BSID II (MDI and PDI)<br><br>**Follow up**<br>• 6 months, 12 months and 18 months<br>• 100% follow-up (only those with complete follow up included)	**MDI at 6 months**<br>Control 88.8±13.4<br>PVE lasting <2weeks: 91.4±14.8<br>PVE persisting >2weeks: 76.5±21.0 *<br><br>**PDI at 6 months**<br>Control 81.7±16.1<br>PVE persisting <2weeks: 84.8±18.0<br>PVE persisting>2weeks: 68.9±18.0*<br><br>**MDI at 12 months**<br>Control 89.0±15.1<br>PVE persisting <2weeks: 90.7±18.1<br>PVE persisting >2weeks: 71.4±17.7*<br><br>**PDI at 12 months**<br>Control 81.7±13.0<br>PVE persisting <2weeks: 76.0±17.2<br>PVE persisting >2weeks: 67.2±18.2*<br><br>**MDI at 18 months**<br>Control 86.2±14.6<br>PVE persisting <2weeks: 85.2±15.0<br>PVE persisting >2weeks: 72.1±21.4*<br><br>**PDI at 18 months**<br>Control 89.7±14.7<br>PVE persisting <2weeks: 83.3±20.2<br>PVE persisting >2weeks: 70.0±24.2*<br>*p<0.001
13	Chmait 2019[29]<br><br>Prospective cohort study<br><br>USA	**Population**<br>• Gestation 24-29 weeks<br>• Monochorionic diamniotic twins treated for twin to twin transfusion syndrome 2007-2010<br><br>**Exposure**:<br>• Cerebral lesion(s) on ultrasound (n=10)<br>• IVH grade 1-2 (n=8)<br>• cPVL (n=2)<br>• Ventriculomegaly/ hydrocephalus (n=1)<br>• Bilateral subependymal cyst (n=1)<br><br>**Comparator**<br>• Unmatched but twin siblings included<br>• No cerebral lesions on ultrasound (n=46)<br><br>**Ascertainment/ definition**<br>• Local database	**Outcomes**<br>• Neurodevelopmental impairment (composite)<br>• Cognitive<br>• Cerebral palsy<br>• Visual impairment<br>• Hearing impairment<br><br>**Assessment/ measurement**<br>• Amiel-Tison examination<br>• Battelle Developmental Inventory 2nd edition<br><br>**Follow-up**<br>• 24 months<br>• 99% follow-up of survivors	**<u>Neurodevelopmental impairment</u>**<br>**Cerebral lesion(s)**<br>n=4; 40%<br>OR 19.28 p<0.001<br><br>**IVH grade 1-2**<br>n=1; 12.5%<br><br>**cPVL**<br>n=2; 100%
14	Choi 2020[30]<br><br>Retrospective case control study<br><br>Korea	**Population**<br>• Admitted to NICU for over 24 hours<br>• Born 2004-2017<br><br>**Exposure**<br>• Germinal matrix hemorrhage (n=15)<br>• Cerebral hemorrhage (n=11)<br><br>**Comparator**<br>• Those with and without hearing loss matched on gender, gestation and birthweight<br>• Infants without germinal matrix or cerebral hemorrhage (figure not specified)<br><br>**Ascertainment/ definition**<br>• National hearing screening program<br>• Medical record review	**Outcomes**<br>• Hearing impairment<br><br>**Assessment/ measurement**<br>• Auditory brainstem response test<br><br>**Follow-up**<br>• 6 months<br>• No follow-up (case control)	**<u>Hearing loss</u>**<br>Germinal matrix hemorrhage n=2, 4.7% p=0.741<br>Cerebral bleeding n=2, 4.7% p=1<br><br>**<u>No hearing loss</u>**<br>Germinal matrix hemorrhage n=13; 7.6%<br>Cerebral bleeding n=9, 5.2%
15<br>\#	Da Silva 2018[31]<br><br>Prospective cohort study<br><br>Brazil	**Population**<br>• Gestation 24-35 weeks<br>• Birth year not specified<br><br>**Exposure**<br>• IVH (n=18)<br><br>**Comparator**<br>• Unmatched but similar gestation and risk of hearing loss<br>• No IVH (n=26)<br><br>**Ascertainment/ definition**<br>• Cranial ultrasound reports verified by neonatologists	**Outcome**<br>• Hearing impairment<br><br>**Assessment/ measurement**<br>• Brainstem auditory evoked potentials<br><br>**Follow-up**<br>• 2-3 months<br>• Completeness of follow-up not specified	**Right ear absolute and interpeak latency: median (min-max)**<br>I: Comparator 1.43 (1.27- 1.67); IVH 1.57 (1.44-2.04) P<0.001<br>III: Comparator 4.00 (3.67-4.30); IVH 4.50 (4.12-5.29) P<0.001<br>V: Comparator 6.33 (5.80-6.73); IVH 6.77 (6.20-8.13) P<0.001<br><br>I-III: Comparator 2.57 (2.00-2.93); IVH 2.84 (2.50-3.70) P<0.001<br>III-V: Comparator 2.27 (2.03-2.80); IVH 2.34 (2.00-2.94) P=0.383<br>I-V: Comparator 4.88 (4.47-5.33); IVH 5.19 (4.68-6.54) P=0.003<br><br>**Left ear absolute and interpeak latency: median (min-max)**<br>I: Comparator 1.48 (1.27-1.97); IVH 1.67 (1.46-1.99) P<0.001<br>III: Comparator 4.07 (3.77-4.57); IVH 4.49 (4.07-5.16) P<0.001<br>V: Comparator 6.32 (5.83-6.80); IVH 6.72 (6.23-8.03) P<0.001<br><br>I-III: Comparator 2.59 (2.17-3.03); IVH 2.81 (2.37-3.57) P=0,011<br>III-V: Comparator 2.30 (1.93-2.90); IVH 2.28 (1.90-3.15) P=0,550<br>I-V: Comparator 4.80 (4.53-5.40); IVH 5.03 (4.56-6.44) P=0,004

SUPPLEMENTAL FIGURE 15
Continued.

16<br># 	DeMauro 2020[32]<br><br>Retrospective cohort study<br><br>USA	**Population**<br>• Gestation < 27 weeks<br>• Birthweight 400-1000g<br>• Born 2010-2014<br>• Had at least one neonatal cranial ultrasound, another cranial ultrasound > 28 days of age<br><br>**Exposure**<br>• IVH grade 1 (n=174)<br>• IVH grade 2 (n=157)<br>• IVH grade 3-4 (n=212)<br>• Ventriculomegaly (n=92)<br>• cPVL or porencephalic cyst (n=169)<br><br>**Comparator**<br>• Unmatched<br>• No abnormalities on cranial ultrasound (n=1502)<br><br>**Ascertainment/ definition**<br>• NICHD neonatal research network<br>• Those with asymmetric findings were classified based on the most severe finding	**Outcomes**<br>• Motor (minor motor abnormalities – composite)<br><br>**Assessment/ measurement**<br>• BSID III<br>• Neurological exam<br>• GMFCS<br>• Parent report<br><br>**Follow-up**<br>• 18-26 months<br>• 73% of survivors	**Motor**<br><br>**Any minor motor finding**<br>IVH 3-4 n=127, 60% aOR 1.4 95%CI (1.02, 1.92)<br>IVH 2 n=84, 54% aOR 1.05 95%CI (0.73, 1.49)<br>IVH 1 n=79, 45% aOR 0.86 95%CI (0.61, 1.2)<br>Comparator n=732, 49%<br><br>Ventriculomegaly n=59, 64% aOR 1.71 95%CI (1.07, 2.72)<br>Comparator n=732, 49%<br><br>cPVL or porencephalic cyst n=113, 67% aOR 1.88 95% CI (1.31, 2.71)<br>Comparator n=732,49%<br><br>**GMFCS level 1**<br>IVH 3-4 n=26, 12% aOR 2.59 95%CI (1.52, 4.43)<br>IVH 2 n=10, 6% aOR 1.68 95%CI (0.82, 3,43)<br>IVH 1 n=6, 3% aOR 0.81 95%CI (0.34, 1.95)<br>Comparator n=62, 4%<br><br>Ventriculomegaly n=14, 15% aOR 3.61 95%CI (1.87, 6.96)<br>Comparator n=62, 4%<br><br>cPVL or porencephalic cyst n=22, 13% aOR 3.29 95%CI (1.86, 5.84)<br>Comparator n=62, 4%<br><br>**BSID III minor motor delay**<br>IVH 3-4 n=92, 43% aOR 0.99 95%CI (0.73, 1.36)<br>IVH 2 n=66, 42% aOR 0.9 95%CI (0.63, 1.29)<br>IVH 1 n=53, 33% aOR 0.7 95%CI (0.49, 0.99)<br>Comparator n=629, 42%<br><br>Ventriculomegaly n=48, 52% aOR 1.43 95%CI (0.91, 2.23)<br>Comparator n=629, 42%<br><br>cPVL or porencephalic cyst n=76, 45% aOR 1.04 95%CI (0.73, 1.48)<br>Comparator n=629, 42%<br><br>**Fine motor score, mean (SD)**<br>IVH 3-4, 7.7 (3.1)<br>IVH 2, 8.3 (3.1)<br>IVH 1, 9.0 (3.0)<br>Comparator, 8.9 (2.7)<br><br>Ventriculomegaly, 8.2 (2.9)<br>Comparator, 8.9 (2.7)<br><br>cPVL or porencephalic cyst, 7.2 (3.4)<br>Comparator, 8.9 (2.7)<br><br>**Gross motor score**<br>IVH 3-4, 6.4 (3.3)<br>IVH 2, 7.1 (2.7)<br>IVH 1, 7.6 (2.6)<br>Comparator, 7.9 (2.5)<br><br>Ventriculomegaly, 6.7 (2.9)<br>Comparator, 7.9 (2.5)<br><br>cPVL or porencephalic cyst, 5.2 (3.4)<br>Comparator, 7.9 (2.5)<br><br>**Any major motor abnormality**<br>IVH 3-4 n=76, 36% aOR 2.83 95%CI (1.99, 4.01)<br>IVH 2 n=30, 19% aOR 1.3 95%CI (0.82, 2.06)<br>IVH 1 n=30, 17% aOR 1.31 95%CI (0.84, 2.04)<br>Comparator n=204, 14%<br><br>Ventriculomegaly n=33, 36% aOR 2.79 95%CI (1.71, 4.54)<br>Comparator n=204, 14%<br><br>cPVL or porencephalic cyst n=99, 59% aOR 8.52 95%CI (5.84, 12.42)<br>Comparator n=204, 14%
17	Duncan 2019[33]<br><br>Retrospective cohort study<br><br>USA	**Population**<br>• Gestation <28 weeks<br>• Born 2005-2009<br>• All had cranial ultrasound and term MRI<br><br>**Exposure**<br>• IVH grade 3-4/cPVL (early cranial ultrasound) (n=39)<br>• IVH grade 3-4/cPVL (late cranial ultrasound) (n=22)<br>• Mild white matter abnormalities (n=233)<br>• Moderate white matter abnormalities (n=61)<br>• Severe white matter abnormalities (n=15)<br>• Cerebellar lesions (n=65)<br><br>**Comparator**<br>• Unmatched<br>• Normal early cranial ultrasound (n=283)	**Outcomes**<br>• Neurodevelopmental impairment (composite)<br>• Cognitive<br>• Motor<br>• Cerebral palsy<br>• Speech and Language<br>• Behavior<br>• Hearing impairment<br>• Visual impairment<br><br>**Assessment/ measurement**<br>• Brief Infant Toddler Social Emotional Assessment (blinded assessment)<br>• BSID III (blinded assessment)<br><br>**Follow-up**<br>• 18-22 months corrected<br>• Follow-up completeness not specified	**Behavior**<br><br>**BITSEA adjusted mean problem scores**<br>IVH 3-4/cPVL (early scan) 12.3 95%CI (9.9-14.6) p=0.801<br>Normal scan 12.6 95%CI (11.4-13.7)<br><br>IVH 3-4/cPVL (late scan) 10.9 95%CI (7.8-14.0)<br>Normal scan 12.9 95%CI (11.7-14.1) p=0.2<br><br>Mild WMA 12.7 95%CI (11.4-14.0) p=0.982<br>Normal scan 12.7 95%CI (11.0-14.4)<br><br>Moderate WMA 12.0 95%CI (9.9-14.0) p=0.534<br>Normal scan 12.7 95%CI (11.0-14.4)<br><br>Severe WMA 12.4 95%CI (8.7-16.0) p=0.857<br>Normal scan 12.7 95%CI (11.0-14.4)<br><br>Cerebellar lesions 13.5 95%CI (11.6-15.5) p=0.339<br>No lesions 12.6 95%CI (11.3-13.8)<br><br>**BITSEA adjusted mean competence scores**<br>IVH 3-4/cPVL (early scan) 17.2 95%CI (15.9-18.5) p=0.283<br>Normal scan 16.5 95%CI (15.8-17.3)

SUPPLEMENTAL FIGURE 15
Continued.

		• Normal late cranial ultrasound (n=287)<br>• Normal MRI (no white matter abnormalities) (n=88)<br>• No lesions on imaging (n=215)<br><br>**Ascertainment/ definition**<br>• NICHD neonatal research network<br>• Images reviewed by blinded central reviewers		IVH 3-4/cPVL (late scan) 17.0 95%CI (15.3-18.6) p=0.799<br>Normal scan 16.8 95%CI (16.0-17.5)<br><br>Mild WMA 16.4 95%CI (15.7-17.2) P=0.272<br>Normal scan 16.9 95%CI (16.0-17.9)<br><br>Moderate WMA 16.8 95%CI (15.7-17.9) P=0.865<br>Normal scan 16.8 95%CI (16.0-17.5)<br><br>Severe WMA 16.1 95%CI (14.2-18.1) P=0.436<br>Normal scan 16.8 95%CI (16.0-17.5)<br><br>Cerebellar lesions 15.7 95%CI (14.6-16.8) p=0.04<br>No lesions 16.8 95%CI (16.1-17.6)
18	Haslam 2018[34]<br><br>Retrospective cohort study<br><br>Canada	**Population (n=2163)**<br>• Gestation 23-28 weeks<br>• Born 2009-2011<br><br>**Exposure**<br>• IVH grade 1-4 (n=798)<br>• IVH grade 3-4 (n=224)<br><br>**Comparison**<br>• Unmatched<br>• No IVH (n=1389)<br>• No IVH grade 3-4 (n=1963)<br><br>**Ascertainment/ definition**<br>• Canadian neonatal network data linked to Canadian neonatal follow-up networks	**Outcomes**<br>• Neurodevelopmental impairment (composite): 7 definitions<br>• Cognitive<br>• Motor<br>• Cerebral palsy<br>• Language<br>• Hearing impairment<br>• Visual impairment<br><br>**Assessment/ measurement**<br>• BSID III<br>• GMFCS<br><br>**Follow-up**<br>• 18-21 months corrected<br>• 93% follow up	**Severe neurodevelopmental impairment**<br>**Lease stringent definition of NDI ((≥1 of: GMFCS 3-5, Bayley-III <−2SD, hearing aid or cochlear implant, bilaterally blind)**<br>IVH (all grades) n=189, 23.7% p<0.01<br>No IVH n=137, 9.9%<br><br>IVH grade 3-4 n=90, 40.2% p<0.01<br>No IVH 3-4 n=236, 11.9%<br>No IVH n=137, 9.9%<br>aOR 4.80 95%CI (3.35-6.87)<br><br>**Most stringent definition of NDI (≥1 of: GMFCS 4-5, Bayley III Cognitive or Language composite score <−3SD, bilaterally blind)**<br>IVH (all grades) n=48, 6% p<0.01<br>No IVH n=29, 2.1%<br><br>IVH grades 3-4 n=32, 14.3% p<0.01<br>No IVH 3-4 n=45, 2.3%<br>aOR 5.54 95%CI (3.27-9.39)
19	Hintz 2015[35]<br><br>Prospective cohort<br><br>USA	**Population**<br>• Gestation 25.9±1.0 weeks<br>• Birth weight 856±190g<br>• Born 2005-2009<br><br>**Exposure** (n=347*)*<br>• White matter abnormalities on MRI (Mild, moderate, and severe)<br>• Any cerebellar lesion<br>• Significant cerebellar lesion<br>• Early adverse cranial ultrasound findings (IVH grade 3-4 or cPVL)<br>• Late adverse cranial ultrasound finding (moderate to severe ventricular enlargement, cPVL, porencephalic cyst or a shunt)<br><br>**Comparator**<br>• Unmatched<br>• Preterm infants<br>• Normal MRI at term<br><br>**Ascertainment/ definition**<br>• Part of a larger NICHD trial<br>• All images reviewed by two blinded pediatric radiologists<br>• Unilateral and bilateral findings grouped together<br>• Papile classification	**Outcomes**<br>• Neurodevelopmental impairment or death (composite)<br>• Neurodevelopmental impairment (composite)<br>• Cognitive<br>• Cerebral palsy (any, moderate, severe)<br>• Significant gross motor impairment<br>• Unimpaired/mildly impaired<br>• Death<br><br>**Assessment/ measurement**<br>• Neurologic exam<br>• GMFCS<br>• BSID III (cognitive score)<br>• Hearing (clinical assessment)<br>• Visual acuity (caregiver report)<br><br>**Follow up**<br>• 18-22 months<br>• 92.7% follow-up	**Neurodevelopmental impairment, n (%)**<br>**White matter injury**<br>Comparator 4 (4.1)<br>Mild white matter abnormalities 16 (6.2)<br>Moderate white matter abnormalities 7 (10.5)<br>Severe white matter abnormalities 11 (61.1)<br>P<0.0001<br><br>**Early cranial ultrasound abnormalities**<br>Comparator 19 (6)<br>All without IVH grade 3-4 or cPVL 26 (6.5)<br>IVH grade 3-4 or cPVL 12 (27.9)<br>P<0.0001<br>**Late cranial ultrasound abnormalities**<br>Comparator 17 (5.4)<br>All without porencephalic cyst, cPVL, moderate to severe ventricular enlargement or in-situ shunt 25 (6)<br>Porencephalic cyst, cPVL, moderate to severe ventricular enlargement or in-situ shunt 13 (50)<br>P<0.0001<br><br>**Neurodevelopmental impairment or death, n (%)**<br>**White matter injury**<br>Comparator 4 (4.1)<br>Mild white matter abnormalities 25 (9.4)<br>Moderate white matter abnormalities 11 (15.5)<br>Severe white matter abnormalities 13 (65)<br>P<0.0001<br><br>**Early cranial ultrasound abnormalities**<br>Comparator 27 (8.3)<br>All without IVH grade 3-4 or cPVL 38 (9.3)<br>IVH grade 3-4 or cPVL 14 (31.1)<br>P<0.0001<br><br>**Late cranial ultrasound abnormalities**<br>Comparator 22 (6.8)<br>All without porencephalic cyst, cPVL, moderate to severe ventricular enlargement or in-situ shunt 38 (8.9)<br>Porencephalic cyst, cPVL, moderate to severe ventricular enlargement or in-situ shunt 15 (53.6)<br>P<0.0001<br><br>**Multivariate logistic regression for neurodevelopmental impairment or death**<br>IVH grade 3-4 or cPVL aOR 0.7 95% CI (0.2, 2.4)<br>Moderate to severe ventricular enlargement or cPVL or porencephalic cyst of shunt in-situ aOR 9.8 95% CI (2.8, 35)<br>Moderate to severe white matter abnormality aOR 1.5 95%CI (0.6, 3.6)<br>Significant cerebellar lesion aOR 3 (1.3, 6.8)<br>(Adjusted for race, late sepsis, BPD, and postnatal steroids)<br><br>**Unimpaired/ mildly impaired, n (%)**<br>**White matter injury**<br>Comparator 69 (70.4)<br>Mild white matter abnormalities 176 (68.2)<br>Moderate white matter abnormalities 40 (59.7)<br>Severe white matter abnormalities 3 (16.7)<br>P<0.0001<br><br>**Early cranial ultrasound abnormalities**<br>Comparator 217 (68)<br>All without IVH grade 3-4 or cPVL 266 (66.8)<br>IVH grade 3-4 or cPVL 22 (51.2)<br>P=0.04

SUPPLEMENTAL FIGURE 15
Continued.

				**Late cranial ultrasound abnormalities**<br>Comparator 220 (69.4)<br>All without porencephalic cyst, cPVL, moderate to severe ventricular enlargement or in-situ shunt 281 (67.7)<br>Porencephalic cyst, cPVL, moderate to severe ventricular enlargement or in-situ shunt 7 (26.9)<br>P<0.0001<br><br>**Cognitive score, mean (SD)**<br>**White matter injury**<br>Comparator 93.5 (14.0)<br>Mild white matter abnormalities 92.6 (13.1)<br>Moderate white matter abnormalities 89.9 (15.3)<br>Severe white matter abnormalities 77.7 (14.5)<br>P<0.0001<br><br>**Early cranial ultrasound abnormalities**<br>Comparator 92.3 (13.5)<br>All without IVH grade 3-4 or cPVL 92.2 (13.7)<br>IVH grade 3-4 or cPVL 88 (16.1)<br>P=0.06<br><br>**Late cranial ultrasound abnormalities**<br>Comparator 92.8 (13.2)<br>All without porencephalic cyst, cPVL, moderate to severe ventricular enlargement or in-situ shunt 92.4 (13.5)<br>Porencephalic cyst, cPVL, moderate to severe ventricular enlargement or in-situ shunt 82 (18.2)<br>P=0.0002<br><br>**Cognitive score <70, n (%)**<br>**White matter injury**<br>Comparator 4 (4.1)<br>Mild white matter abnormalities 11 (4.3)<br>Moderate white matter abnormalities 7 (10.5)<br>Severe white matter abnormalities 4 (22.2)<br>P=0.011<br><br>**Early cranial ultrasound abnormalities**<br>Comparator 16 (5)<br>All without IVH grade 3-4 or cPVL 21 (5.3)<br>IVH grade 3-4 or cPVL 5 (11.6)<br>P=0.16<br><br>**Late cranial ultrasound abnormalities**<br>Comparator 13 (4.1)<br>All without porencephalic cyst, cPVL, moderate to severe ventricular enlargement or in-situ shunt 20 (4.8)<br>Porencephalic cyst, cPVL, moderate to severe ventricular enlargement or in-situ shunt 6 (23.1)<br>P=0.0024<br><br>**Cognitive score <85, n (%)**<br>**White matter injury**<br>Comparator 20 (20.4)<br>Mild white matter abnormalities 47 (18.2)<br>Moderate white matter abnormalities 20 (29.9)<br>Severe white matter abnormalities 11 (61.1)<br>P<0.0001<br><br>**Early cranial ultrasound abnormalities**<br>Comparator 65 (20.4)<br>All without IVH grade 3-4 or cPVL 83 (20.9)<br>IVH grade 3-4 or cPVL 15 (34.9)<br>P=0.04<br><br>**Late cranial ultrasound abnormalities**<br>Comparator 60 (18.9)<br>All without porencephalic cyst, cPVL, moderate to severe ventricular enlargement or in-situ shunt 84 (20.2)<br>Porencephalic cyst, cPVL, moderate to severe ventricular enlargement or in-situ shunt 14 (53.9)<br>P<0.0001<br><br>**Any cerebral palsy, n (%)**<br>**White matter injury**<br>Comparator 2 (2)<br>Mild white matter abnormalities 14 (5.4)<br>Moderate white matter abnormalities 4 (5.9)<br>Severe white matter abnormalities 11 (61.1)<br>P<0.0001<br><br>**Early cranial ultrasound abnormalities**<br>Comparator 9 (2.8)<br>All without IVH grade 3-4 or cPVL 17 (4.2)<br>IVH grade 3-4 or cPVL 14 (32.6)<br>P<0.0001<br><br>**Late cranial ultrasound abnormalities**<br>Comparator 11 (3.4)<br>All without porencephalic cyst, cPVL, moderate to severe ventricular enlargement or in-situ shunt 17 (4.1)<br>Porencephalic cyst, cPVL, moderate to severe ventricular enlargement or in-situ shunt 14 (53.9)<br>P<0.0001<br><br>**Moderate to severe cerebral palsy, n (%)**<br>**White matter injury**<br>Comparator 0 (0)<br>Mild white matter abnormalities 3 (1.2)<br>Moderate white matter abnormalities 1 (1.5)

SUPPLEMENTAL FIGURE 15

Continued.

				Severe white matter abnormalities 9 (50)<br>P<0.0001<br><br>**Early cranial ultrasound abnormalities**<br>Comparator 2 (0.6)<br>All without IVH grade 3-4 or cPVL 5 (1.2)<br>IVH grade 3-4 or cPVL 8 (18.6)<br>P<0.0001<br><br>**Late cranial ultrasound abnormalities**<br>Comparator 1 (0.3)<br>All without porencephalic cyst, cPVL, moderate to severe ventricular enlargement or in-situ shunt 4 (1)<br>Porencephalic cyst, cPVL, moderate to severe ventricular enlargement or in-situ shunt 9 (34.6)<br>P<0.0001<br><br>**Significant gross motor impairment, n (%)**<br>**White matter injury**<br>Comparator 1 (1)<br>Mild white matter abnormalities 5 (1.9)<br>Moderate white matter abnormalities 1 (1.5)<br>Severe white matter abnormalities 10 (55.6)<br>P<0.0001<br><br>**Early cranial ultrasound abnormalities**<br>Comparator 4 (1.2)<br>All without IVH grade 3-4 or cPVL 8 (2)<br>IVH grade 3-4 or cPVL 9 (20.9)<br>P<0.0001<br><br>**Late cranial ultrasound abnormalities**<br>Comparator 4 (1.3)<br>All without porencephalic cyst, cPVL, moderate to severe ventricular enlargement or in-situ shunt 7 (1.7)<br>Porencephalic cyst, cPVL, moderate to severe ventricular enlargement or in-situ shunt 10 (38.5)<br>P<0.0001<br><br>**Multivariate logistic regression for gross motor impairment or death**<br>IVH grade 3-4 or cPVL aOR 0.8 95% CI (0.2, 3.3)<br>Moderate to severe ventricular enlargement or cPVL or porencephalic cyst of shunt in-situ aOR 10.9 95% CI (2.5, 47.3)<br>Moderate to severe white matter abnormality aOR 1.6 95%CI (0.5, 4.9)<br>Significant cerebellar lesion aOR 5.2 (1.9, 14.1)<br>(Adjusted for race, multiple gestation, maternal insurance, late sepsis, BPD, and postnatal steroids).
20	Klebermass-Schrehof 2012[36]<br><br>Prospective cohort<br><br>Austria	**Population**<br>• Gestation <32 weeks<br>• Admitted to NICU 1994-2005<br><br>**Exposure**<br>• IVH grade 1 (n=37)<br>• IVH grade 2 (n=84)<br>• IVH grade 3 (n=18)<br>• IVH grade 4 (n=12)<br><br>**Comparator**<br>• Unmatched<br>• No IVH (n=320)<br><br>**Ascertainment/ definition**<br>• Papile classification<br>• DeVries classification<br>• Most severe lesion used	**Outcomes**<br>• Cognitive<br>• Language<br>• Motor<br>• Visual<br>• Cerebral palsy<br>• Neurosensory impairment (composite)<br><br>**Measurement/assessment**<br>• BSID II (MDI, PDI)<br>• K-ABC<br>• Beery-Buktenica Developmental Test of Visual-Motor Integration<br>• Clinical assessment<br><br>**Follow-up**<br>• 1 and 2 years (3.5, 5 years)<br>• Only included those with follow-up	**Infants born <32 weeks' gestation**<br>**Outcome at 1 year**<br>**PDI <70**<br>IVH grade 1 n=12; 42.3%<br>IVH grade 2 n=39; 45.8%<br>No IVH n=45; 15%<br><br>**PDI mean +/-SD**<br>IVH grade 1 76.1+/-20.7 P.0.005<br>IVH grade 2 70.8+-18.3 p<0.0001<br>No IVH 85.7+/-16.8<br><br>**MDI <70**<br>IVH grade 1 n=2; 7.6%<br>IVH grade 2 n=22; 25.7%<br>No IVH n=29; 9.7%<br><br>**MDI mean +/-SD**<br>IVH grade 1 86.3+/-11.2 p not significant<br>IVH grade 2 79.5+/-18.1 p=0.003<br>No IVH 89.7+/-14.5<br><br>**Outcomes at 2 years**<br>**PDI <70**<br>IVH grade 1 n=;5 18.2%<br>IVH grade 2 n=22; 26.4%<br>No IVH n=39; 12.9%<br><br>**PDI mean +/-SD**<br>IVH grade 1 84.5+/-16 not significant<br>IVH grade 2 84.4+/-20.6 p=0.02<br>No IVH 90.3+/-17.1<br><br>**MDI <70**<br>IVH grade 1 n=8; 27.3%<br>IVH grade 2 n=20; 24.1%<br>No IVH n=36; 11.8%<br><br>**MDI mean +/-SD**<br>IVH grade 1 83.8+/-19.5 p=0.03<br>IVH grade 2 86.5+/-20.1 not significant<br>No IVH 91 +/-18.3<br><br>**Infants born <28 weeks' gestation**<br>**Outcomes at 1 year**<br>**PDI <70**<br>IVH grade 3 77.8 %<br>IVH grade 4 90 %<br>No IVH 16.2 %<br><br>**PDI mean (SD)**<br>IVH grade 3 59.3 (19.9) P<0.01

SUPPLEMENTAL FIGURE 15
Continued.

				IVH grade 4 53.6 (11.4) P<0.01<br>No IVH 84.6 (17.0)<br><br>**MDI <70**<br>IVH grade 3 78.8 %<br>IVH grade 4 66.7 %<br>No IVH 10.6%<br><br>**MDI mean (SD)**<br>IVH grade 3 63.0 (23.3) P<0.01<br>IVH grade 4 59.0 (15.7) P<0.01<br>No IVH 8.9 (15.1)<br><br>**Outcomes at 2 years**<br>**PDI <70**<br>IVH grade 3 75%<br>IVH grade 4 66.7%<br>No IVH 13.4%<br><br>**PDI mean (SD)**<br>IVH grade 3 61.8 (21.9) p<0.01<br>IVH grade 4 62.1 (16.2) p<0.01<br>No IVH 89.8 (16.5)<br><br>**MDI <70**<br>IVH grade 3 75 %<br>IVH grade 4 50%<br>No IVH 11.4%<br><br>**MDI mean (SD)**<br>IVH grade 3 62.1 (23.6) p<0.01<br>IVH grade 4 68.1 (21.3) p<0.01<br>No IVH 90.0 (17.8)<br><br>**Outcomes at 5.5 years**<br>**KABC <70**<br>No IVH 5.2 %<br>IVH grade 1 5.9 %<br>IVH grade 2 10.5 %<br><br>**KABC mean (SD)**<br>No IVH 94.1(14.8)<br>IVH grade 1 95.4 (16.1) P= not significant<br>IVH grade 2 88.2 (15.8) p=0.02<br><br>**Cerebral palsy**<br>No IVH 11.4 %<br>IVH grade 1 22.2 % p=0.02<br>IVH grade 2 47.4 % p<0.0001<br><br>**Hearing impairment**<br>No IVH 1.7%<br>IVH grade 1 0% P= not significant<br>IVH grade 2 2.5% P= not significant<br><br>**Visual impairment**<br>No IVH 5.9 %<br>IVH grade 1 17.9 % p=0.01<br>IVH grade 2 21% p=0.002<br><br>**Visuomotor integration mean (SD)**<br>No IVH 97.8 (19.5)<br>IVH grade 1 94 (16.1) P= not significant<br>IVH grade 2 92.5 (15.4) P= not significant
21	Kratimenos 2019[37]<br><br>Retrospective cohort study<br><br>USA	**Population**<br>• Gestation <37 weeks<br>• Admitted to NICU 2010-2015<br><br>**Exposure**<br>• IVH grade 1-4 (no figures)<br><br>**Comparator**<br>• Unmatched<br>• No IVH (no figures)<br><br>**Ascertainment/ definition**<br>• Not specified	**Outcomes**<br>• Motor<br>• Speech and language<br><br>**Assessment/ measurement**<br>• REEL 2 score<br><br>**Follow-up**<br>• 30 months corrected<br>• Completeness of follow-up not specified	**Sensory development**<br>**IVH**<br>Univariate 95% CI [–1.31, 0.08] p= 0.0857<br><br>**Motor development**<br>**IVH**<br>Univariate 95% CI [–1.3, 0.1] p=0.0956<br><br>**Fine motor development**<br>**IVH**<br>Univariate 95% CI [–6.72, -3.54] p<0.0001<br>Multivariate 95% CI [–6.48, -3.23] p<0.0001<br><br>**Gross motor development**<br>**IVH**<br>Univariate 95% CI [–5.97, -2.71] p<0.0001<br>Multivariate 95% CI [–5.78, -2.4] p<0.0001
22	Lean 2019[38]<br><br>Retrospective cohort study<br><br>USA	**Population**<br>• Gestation ≤30 weeks<br>• Birth year not specified<br><br>**Exposure**<br>• IVH grade 3-4, and/or post haemorrhagic hydrocephalus or cPVL (n=27)<br><br>**Comparator**<br>• Unmatched<br>• Preterm infants (similar gestation to exposed group)<br>• No brain injury on cranial ultrasound or MRI (n=59)<br><br>**Ascertainment/ definition**	**Outcomes**<br>• Cognitive<br>• Motor<br>• Speech and language<br>**Assessment/ measurement**<br>• BSID III<br>**Follow-up**<br>• 2 years<br>• 84-86% follow-up	**Cognitive**<br>**Cognitive scores: BSID III, mean (95% CI)**<br>Brain injury: 80.96 (76.65– 85.26)<br>Comparator: 86.61 (83.75–89.47)<br>OR 0.49 p=0.03<br>Adjusted p=0.16<br><br>**Cognitive delay (BSID III <85)**<br>Brain injury: n=14, 50%<br>Comparator: n=18, 30.5%<br>OR 2.28 p=0.09<br>Adjusted p=0.18<br><br>**Language**<br>**Language scores: BSID III, mean (95% CI)**<br>Brain injury: 82.85 (77.97–87.73)<br>Comparator: 89.81 (86.51–93.10)<br>OR 0.56 p=0.02

SUPPLEMENTAL FIGURE 15
Continued.

		• Images reviewed by pediatric radiologist<br>• Papile classification		Adjusted p=0.11<br>**Language delay (BSID III <85)**<br>Brain injury: n=15, 53.8%<br>Comparator: n=19, 31.6%<br>OR 2.53 p=0.05<br>Adjusted p=0.11<br><br>**Motor**<br>**Motor scores: BSID III, mean (95% CI)**<br>Brain injury: 72.22 (67.55–76.89)<br>Comparator: 85.59 (82.40–88.77)<br>OR 1.07 p<0.001<br>Adjusted p=0.001<br><br>**Motor delay (BSID III <85)**<br>Brain injury: n=21, 77.8%<br>Comparator: n=17, 29.3%<br>OR 8.44 p<0.001<br>Adjusted p=0.001
23	Lin 2020[39]<br><br>Prospective cohort study<br><br>Taiwan	**Population**<br>• Birthweight ≤ 1500g<br>• Born 2002-2009<br><br>**Exposure**<br>• IVH grade 3-4 (n=175)<br>• cPVL n=170<br><br>**Comparator**<br>• Unmatched<br>• No further details provided<br><br>**Ascertainment/ definition**<br>• Taiwan premature infant follow-up network database	**Outcomes**<br>• Overall disability (composite)<br>• Cognitive<br>• Motor<br>• Vision<br>• Hearing<br><br>**Assessment/ measurement**<br>• BSID II or BSID III<br>• WPPSI-R<br><br>**Follow-up**<br>• 2 years; 76% follow-up<br>• 5 years; 26% follow-up	**Abnormal neurodevelopmental outcome / moderate to severe neurodevelopmental disorder**<br>**2 years**<br>IVH OR a2.90 95% CI (1.66-5.04) P= 0.0002<br>cPVL OR a5.08 95% CI (3.06-8.45)P <0.0001*<br><br>**2-5 years**<br>IVH aOR 0.68 95% CI (0.18-2.51) P=0.56<br>cPVL aOR 8.12 95% CI (6.11-53.72) P<0.0001*<br><br>**5 years**<br>IVH aOR 1.35 95% CI (0.39-4.64) P=0.64<br>cPVL aOR 6.76 95% CI (6.86-40.94) P<0.0001
24	Logan 2011[40]<br><br>Retrospective cohort<br>USA	**Population**<br>• Gestation <28 weeks<br>• Born 2002-2004<br><br>**Exposure**<br>Indicators of white matter damage including:<br>• Moderate to severe ventriculomegaly (n=105)<br>• Echolucent lesion (n=73)<br>• (Not mutually exclusive groups)<br><br>**Comparator**<br>• Unmatched<br>• Preterm infants<br>• No ventriculomegaly (n=936)<br>• No echolucent lesion (n=968)<br>• (Not mutually exclusive groups)<br><br>**Ascertainment/ definition**<br>• ELGAN study<br>• Maternal interview<br>• Medical record review<br>• Imaging reviewed by two blinded sonographers	**Outcomes**<br>• Cerebral palsy<br><br>**Measurement/ assessment**<br>• Structured neurological exam<br>• Previously published algorithm for cerebral palsy types<br><br>**Follow up**<br>• 24 months<br>• 86.4% follow-up	**Cerebral palsy**<br>**Hemiparesis**<br>Ventriculomegaly (n=9, 9%)<br>No ventriculomegaly (n=9, 1%)<br><br>Echolucent lesion (n=9, 12%)<br>No echolucent lesion (n=10, 1%)<br><br>**Diparesis**<br>Ventriculomegaly (n=9, 9%)<br>No ventriculomegaly (n=28, 3%)<br><br>Echolucent lesion (n=5, 7%)<br>No echolucent lesion (n=29, 3%)<br><br>**Quadriparesis**<br>Ventriculomegaly (n=29, 28%)<br>No ventriculomegaly (n=37, 4%)<br><br>Echolucent lesion (n=24, 33%)<br>No echolucent lesion (n=39, 4%)
25	Matsushita 2019[41]<br><br>Retrospective Case control<br><br>Japan	**Population (n=8431)**<br>• Birthweight <1500g<br>• Born 2003-2012<br><br>**Exposure**<br>• IVH grade 3-4 (figures not specified)<br>• cPVL (figures not specified)<br><br>**Comparator**<br>• Not specified<br><br>**Ascertainment/ definition**<br>• Neonatal research network database (Japan)<br>• Papile classification	**Outcomes**<br>• Cognitive<br>• Motor<br>• Cerebral palsy<br>• Epilepsy<br><br>**Assessment/ measurement -** trained testers<br>• Kyoto Scale of Psychological Development<br>• Enjoji Scale of Infant Analytical Development<br><br>**Follow-up**<br>• 3 years<br>• 100% (only those with complete epilepsy follow-up data were included)	**Association with epilepsy (odds of below neonatal events in those with epilepsy)**<br>**IVH grade 3-4**<br>OR 17.1 95% CI (11.6, 25.3) p<0.01<br>aOR 5.13 95% CI (2.10, 12.5) p<0.01<br><br>**cPVL**<br>OR 18.3 95% CI (12.6, 26.7) p<0.01<br>aOR 12.7 95% CI (5.34, 30.3) p<0.01<br><br>**Factors associated any neurological sequelae (epilepsy, psychomotor delay or cerebral palsy)**<br>**IVH grade 3-4**<br>OR 10 95% CI (8.32, 15.1) p<0.01<br>aOR 6.15 95% CI (3.46, 10.9) p<0.01<br><br>**cPVL**<br>OR 15.5 95% CI (11.5, 21) p<0.01<br>aOR 13 95% CI (6.53, 26) p<0.01<br><br>**Factors associated with all three neurological sequelae (epilepsy, psychomotor delay and cerebral palsy)**<br>**IVH grade 3-4**<br>OR 13.6 95% CI (6.73, 27.5) p<0.01<br>aOR 11.6 95% CI (2.32, 57.5) p<0.01<br><br>**cPVL**<br>OR 21.8 95% CI (11.5, 41.4) p<0.01<br>aOR 10.2 95% CI (2.22, 47.2) p<0.01
26	Miller 2005[42]<br><br>Prospective cohort<br><br>USA	**Population**<br>• Gestation <34 weeks<br>• Born 1998-2003<br><br>**Exposure**<br>• White matter injury (minimal, moderate, severe) (n=41)<br>• Ventriculomegaly (n=14)<br>• IVH grade 1-2 (n=24)<br>• IVH grade 3-4 (n=6)	**Outcome**<br>• Neurodevelopmental outcome (composite)<br><br>**Assessment/ measurement**<br>• BSID II (MDI)<br>• Neurological exam<br><br><br>**Follow-up**	**Abnormal neurodevelopmental outcome**<br>**First MRI**<br>**White matter injury, n (%)**<br>None 4 (33%)<br>Minimal 1 (8%)<br>Moderate 5 (42%)<br>Severe 2 (17%)<br>P=0.03<br><br>**Ventriculomegaly**

SUPPLEMENTAL FIGURE 15
Continued.

		• Cerebellar hemorrhage on MRI (n=9)<br>• Moderate to severe MRI abnormalities (moderate to severe white matter injury, any ventriculomegaly or IVH grade 3) (n=32)<br><br>**Control**<br>• Unmatched<br>• Respective injury type not present on MRI (overlap unclear)<br><br>**Ascertainment/ definition**<br>• MRI imaging reviewed by two blinded pediatric neuroradiologists<br>• Papile classification	• 12-18 months<br>• 89% follow-up	None 6 (46%)<br>Mild 2 (15%)<br>Moderate/severe 5 (38%)<br>P<0.0001<br><br>**Intraventricular hemorrhage**<br>None 5 (38%)<br>Grade 1-2 5 (38%)<br>Grade 3-4 3 (23%)<br>Cerebellar hemorrhage 0<br>P=0.006<br><br>**<u>Second MRI</u>**<br>**White matter injury**<br>None 2 (29%)<br>Minimal 0 (0%)<br>Moderate 4 (57%)<br>Severe 1 (14%)<br>P=0.05<br><br>**Ventriculomegaly**<br>None 4 (57%)<br>Mild 1 (14%)<br>Moderate/severe 2 (29%)<br>P=0.003<br><br>**Intraventricular hemorrhage**<br>None 3 (43%)<br>Grade 1-2 3 (43%)<br>Grade 3 1 (14%)<br>Cerebellar hemorrhage 0<br>P=0.005
27	Nair 2021[43]<br><br>Case control study<br><br>England	**Population**<br>• Gestation ≤ 32 weeks<br>• Born 2005-2019<br>• Admitted to NICU for over 48 hours<br><br>**Exposure**<br>• IVH grade 3-4<br><br>**Comparator**<br>• Matched on outcome (case-control)<br>• No IVH grade 3-4<br><br>**Ascertainment/ definition**<br>• National information technology system for the newborn hearing screening program<br>• Neonatal database<br>• Case notes<br>• Hospital electronic systems	**Outcomes**<br>• Hearing impairment (unilateral and bilateral)<br><br>**Assessment/ measurement**<br>• Newborn hearing screening<br><br>**Follow-up**<br>• 2-17 months<br>• No follow-up (case-control)	**<u>Hearing loss</u>**<br>IVH grade 3-4 n=8. 36.4%<br><br>**<u>No hearing loss</u>**<br>IVH grade 3-4 n=5, 5.4%<br>OR 10 95% CI 2.9, 35 p<0.001*<br>aOR 14 95% CI 0.7, 286.6 p=0.08
28	Patra 2006[44]<br><br>Prospective cohort<br><br>USA	**Population**<br>• Extremely low birth weight infants (<1000g) without congenital malformations<br>• Born 1992-2000<br><br>**Exposure**<br>• IVH grade 1-2 (n=104)<br><br>**Comparison**<br>• Unmatched<br>• Normal cranial ultrasound (n=258)<br><br>**Ascertainment/ definition**<br>• Papile classification<br>• Most severe imaging finding used	**Outcomes**<br>• Major neurologic abnormality<br>• Cerebral palsy<br>• Deafness<br>• Cognitive<br>• Motor<br>• Neurodevelopmental impairment (composite)<br><br>**Measurement/assessment**<br>• Physical exam<br>• Neurological exam of tone (Amiel-Tison)<br>• BSID II (MDI, PDI)<br><br>**Follow-up**<br>• 20 months<br>• 91% follow-up	**Major neurological abnormality (* p<0.05)**<br>Grade 1-2 IVH (n=13; 13%)<br>Comparison (n=14; 5%)<br><br>**Cerebral palsy**<br>Grade 1-2 IVH (n=8; 8%)<br>Comparison (n=9; 3%)<br><br>**Hypertonia**<br>Grade 1-2 IVH (n=3; 3%)<br>Comparison (n=3; 1%)<br><br>**Hypotonia**<br>Grade 1-2 IVH (n=2; 2%)<br>Comparison (n=2; 1%)<br><br>**Deafness (unilateral or bilateral)* (p<0.05)**<br>Grade 1-2 IVH (n=9; 9%)<br>Comparison (n=6; 2%)<br><br>**<u>Mental Developmental Index (MDI)</u>**<br>**Mean +/-SD score**<br>Grade 1-2 IVH 74 +/-16*(p<0.01)<br>Comparison 79+/-14<br><br>**MDI<70**<br>Grade 1-2 IVH (n=47; 45%)* (p<0.01)<br>Comparison (n=65; 25%)<br><br>**<u>Psychomotor Development Index (PDI)</u>**<br>**Mean +/-SD score**<br>Grade 1-2 IVH 74+/-16* (p<0.05)<br>Comparison 77+/-15<br><br>**PDI<70**<br>Grade 1-2 IVH (n=36; 35%)<br>Comparison (n=63; 28%)<br><br>**Neurodevelopmental impairment +/- MDI <70**<br>Grade 1-2 IVH (n=49; 47%)** (p<0.001)<br>Comparison (n=72; 28%)<br><br>**<u>Multivariate regression of neurosensory and developmental outcomes</u>**<br>**Grade 1-2 IVH versus comparison group**<br><br>MDI<70 aOR 2 (1.2, 3.3)**

SUPPLEMENTAL FIGURE 15
Continued.

				Major neurological abnormality aOR 2.6 (1.06, 6.36)*<br><br>Neurodevelopmental impairment aOR 1.83 (1.11, 3.03)*.
29	Pavaine 2016[45]<br><br>Prospective cohort<br><br>Canada	**Population**<br>• Gestation 24-33 weeks<br>• Year of birth not reported<br><br>**Exposure (n=44*)***<br>• Mild to moderate brain injury: isolated punctate white matter lesions +/- IVH grade 1-3<br>• Severe injury: confluent white matter lesions ± destructive lesions<br><br>**Comparator**<br>• Unmatched<br>• No brain injury on imaging (n=41)<br><br>**Ascertainment/ definition**<br>• Papile and Volpe classifications adapted for MRI<br>• Imaging independently assessed by two blinded pediatric neuroradiologists	**Outcomes**<br>• Motor<br>• Vision<br><br>**Assessment/ measurement**<br>• BSID III (2 years)<br>• Beery Buktenica (4 years)<br><br>**Follow-up**<br>• 2 years: 48% follow-up<br>• 4 years: 51.8% follow-up	**Bayley motor: n, mean (SD) range**<br>No injury: 22, 94.7 (11.9) 76-121<br>Mild to moderate injury: 15. 89.3 (11.2) 64-107<br>Severe injury: 4, 95.8 (12.7) 79-110<br><br>**Beery visual-motor integration n, mean (SD) range**<br>No injury 20 98.9 (8.8) 85–121<br>Mild/moderate injury 19 97.1 (12.8) 66–119<br>Severe injury 5 103 (13.5) 88–119<br><br>**Beery visual perception n, mean (SD) range**<br>No injury 19 93.1 (18.9) 52–115<br>Mild/moderate injury 18 90.9 (22.5) 46–136<br>Severe injury 5 91.2 (21.8) 74–117<br><br>**Beery motor coordination n, mean (SD) range**<br>No injury 19 85.6 (16.8) 58–118<br>Mild/moderate injury 18 82.7 (14.6) 67–126<br>Severe injury 5 87.6 (16.6) 68–105
30	Payne 2013[46]<br><br>Prospective cohort<br><br>USA	**Population**<br>• Gestation <27 weeks<br>• Born 2006-2008<br><br>**Exposure (n=451)**<br>• IVH grade 1-2 (n=270)<br>• IVH grade 3-4 (n=181)<br><br>**Comparator (n=1021)**<br>• Unmatched<br>• No IVH on cranial ultrasound<br><br>**Ascertainment/ definition**<br>• Papile classification<br>• Classified according to most severe injury.	**Outcomes**<br>• Cerebral palsy<br>• Motor<br>• Visual impairment<br>• Hearing impairment<br>• Cognitive<br>• Language<br>• Neurodevelopmental impairment (composite)<br><br>**Assessment/ measurement**<br>• Medical history<br>• Neurological exam<br>• Developmental assessment<br>• Behavioral tests<br>• GMFCS<br>• BSID III<br><br>**Follow up**<br>• 18-22 months<br>• 87.4% follow-up	**Any cerebral palsy**<br>No IVH (n=82, 8%)<br>Grade 1-2 IVH (n=24, 9%)<br>Grade 3-4 IVH (n=51, 28%)<br><br>Odds of any cerebral palsy with grade 1-2 IVH compared to no IVH aOR 1 (0.61, 1.64)<br>Odds of any cerebral palsy with grade 3-4 IVH compared to no IVH aOR 3.43 (2.24, 5.27)<br><br>**Moderate to severe cerebral palsy**<br>No IVH (n=41, 4%)<br>Grade 1-2 PIVH (n=5, 2%)<br>Grade 3-4 PIVH (n=18, 10%)<br><br>**Gross motor functional limitation**<br>No IVH (n=51, 5%)<br>Grade 1-2 IVH (n=8, 3%)<br>Grade 3-4 IVH (n=25, 14%)<br><br>Odds of GMFCS >2 with grade 1-2 IVH compared to no IVH aOR 0.66 (0.32, 1.39)<br>Odds of GMFCS >2 with grade 3-4 IVH compared to no IVH aOR 2.51 (1.43, 4.44)<br><br>**Severe visual impairment**<br>No IVH (n=10, 1%)<br>Grade 1-2 IVH (n=3, 1%)<br>Grade 3-4 IVH (n=2, 1%)<br><br>**Deafness**<br>No IVH (n=31, 3%)<br>Grade 1-2 IVH (n=8, 3%)<br>Grade 3-4 IVH (n=4, 2%)<br><br>**Cognitive score, mean score (SD)**<br>No IVH 90 (14)<br>Grade 1-2 IVH 89 (14)<br>Grade 3-4 IVH 84 (15)<br><br>Adjusted regression coefficient (B) for Grade 1-2 IVH compared to no IVH -0.54 (-2.34,1.25)<br>Adjusted regression coefficient (B) for Grade 3-4 IVH compared to no IVH -4.46 (-6.62 to -2.30)<br><br>**Cognitive score <70**<br>No IVH (n=71, 7%)<br>Grade 1-2 IVH (19, 7%)<br>Grade 3-4 IVH (27, 15%)<br>Odds of cognitive score <70 with grade 1-2 IVH compared to no IVH aOR 0.94 (0.54, 1.61)<br>Odds of cognitive score <70 with grade 3-4 IVH compared to no IVH aOR 1.37 (0.79, 2.37)<br><br>**Cognitive score <85**<br>No IVH (n=255, 25%)<br>Grade 1-2 IVH (n=78, 29%)<br>Grade 3-4 IVH (n=80, 44%)<br>Odds of cognitive score <85 with grade 1-2 IVH compared to no IVH aOR 1.03 (0.75, 1.43)<br>Odds of cognitive score <85 with grade 3-4 IVH compared to no IVH aOR 1.82 (1.26, 2.64)<br><br>**Language score, mean (SD)**<br>No PIVH 86 (17)<br>Grade 1-2 IVH 83 (15)<br>Grade 3-4 IVH 80 (18)<br>Adjusted regression coefficient (B) for Grade 1-2 IVH compared to no IVH -0.31 (-2.45, 1.83)<br>Adjusted regression coefficient (B) for Grade 3-4 IVH compared to no IVH -3.50 (-6.10, -0.90)<br><br>**Language score <70**<br>No IVH (n=163, 16%)<br>Grade 1-2 IVH (n=43, 16%)<br>Grade 3-4 IVH (n=52, 29%)<br><br>Odds of language score <70 with grade 1-2 IVH compared to no IVH aOR 0.76 (0.52, 1.13)<br>Odds of language score <70 with grade 3-4 IVH compared to no IVH aOR 1.57 (1.04, 2.37)<br><br>**Language score <85**<br>No IVH (n=459, 45%)

SUPPLEMENTAL FIGURE 15
Continued.

				Grade 1-2 IVH (n=143, 53%)<br>Grade 3-4 IVH (n=107, 59%)<br><br>Odds of language score <85 with grade 1-2 IVH compared to no IVH aOR 1.08 (0.80, 1.45)<br>Odds of language score <85 with grade 3-4 IVH compared to no IVH aOR 1.45 (1.00, 2.10)<br><br>**Neurodevelopmental impairment with cognitive score <70**<br>No IVH (n=102, 10%)<br>Grade 1-2 IVH (n=27, 10%)<br>Grade 3-4 IVH (n=40, 22%)<br><br>Odds of neurodevelopmental impairment with cognitive score <70 with grade 1-2 IVH compared to no IVH aOR 0.82 (0.51, 1.31)<br>Odds of neurodevelopmental impairment with cognitive score <70 with grade 3-4 IVH compared to no IVH aOR 1.68 (1.06, 2.65)<br><br>**Neurodevelopmental impairment with cognitive score <85**<br>No IVH (n=276, 27%)<br>Grade 1-2 IVH (n=81, 30%)<br>Grade 3-4 IVH (n=83, 46%)<br><br>Odds of neurodevelopmental impairment with cognitive score <85 with grade 1-2 IVH compared to no IVH aOR 1.00 (0.73, 1.37)<br>Odds of neurodevelopmental impairment with cognitive score <85 with grade 3-4 IVH compared to no IVH aOR 1.78 (1.24, 2.57)
31	Peixoto 2018[47]<br><br>Retrospective cohort study<br><br>Portugal	**Population**<br>• Gestation <34 weeks<br>• Admitted to NICU 2006-2015<br><br>**Exposure**<br>• IVH grade 1-2 (n=86)<br><br>**Comparison**<br>• Matched 1:1 on gestation and year of birth<br>• No IVH on cranial ultrasound (n=86)<br><br>**Ascertainment/ definition**<br>• Papile classification<br>• Classified according to most severe injury	**Outcomes**<br>• Neurodevelopmental delay (composite)<br>• Cognitive<br>• Motor<br>• Cerebral palsy<br>• Vision<br>• Hearing<br>• Social<br><br>**Assessment/ measurement**<br>• Neurological exam (based on Amiel-Tison)<br>• Hearing testing<br>• Vision testing<br>• Developmental testing<br>• GMFCS<br>• Griffiths Mental Development Scales<br><br>**Follow-up**<br>• 24 months<br>• Completeness of follow-up not specified	**Severe neurodevelopmental delay (composite)**<br>IVH 1-2 n=3, 3.5%<br>Comparator n=1, 1.2% p=0.317<br><br>**Cognitive**<br>**Global developmental quotient mean +/-SD**<br>IVH 1-2 94.4 ± 12.7<br>Comparator 98.6 ± 9.8 p=0.02<br><br>**Global developmental quotient <70**<br>IVH 1-2 n=2, 2.3%<br>Comparator n=1, 1.2% p= 0.567<br><br>**Cerebral palsy**<br>IVH 1-2 n=1, 1.2%<br>Comparator n=0, 0% p= 0.993<br><br>**Visual impairment**<br>IVH 1-2 n=-0, 0%<br>Comparator n=0, 0%<br><br>**Hearing impairment**<br>IVH 1-2 n=-1, 1.2%<br>Comparator n=1, 1.2% p=0.993
32	Radic 2015[48]<br><br>Prospective cohort<br><br>Canada	**Population**<br>• Gestation ≤30 weeks<br>• Born 1993-2010<br><br>**Exposure**<br>• IVH grade 1-4 (n= 370)<br>• IVH grade 1 (n=145)<br>• IVH grade 2 (n=62)<br>• IVH grade 3 (n=42)<br>• IVH grade 4 (n=39)<br><br>**Comparator**<br>• Unmatched<br>• No IVH (n=830)<br><br>**Ascertainment/ definition**<br>• Nova Scotia database<br>• Papile classification<br>• Mixture of ultrasound and MRI used	**Outcomes**<br>• Disability (composite)<br>• Cognitive<br>• Language<br>• Cerebral palsy<br>• Bilateral blindness<br>• Bilateral deafness<br>• Mortality<br><br>**Measurement/ assessment**<br>• BSID II (MDI)<br>• BSID III<br><br>**Follow-up**<br>• 2-3 years<br>• 85% followed-up (13% died)	**Death**<br>No IVH (n=81; 10%)<br>IVH Grade 1 (n=11; 7%) RR 0.71 (p 0.259)<br>IVH Grade 2 (n=14; 18%) RR 1.84 (p 0.024)<br>IVH Grade 3 (n=15; 26%) RR 2.7 (p <0.001)<br>IVH Grade 4 (n=36; 47%) RR 4.85 (p <0.001)<br><br>**Disability**<br>Grade 0 (n=175;24%)<br>Grade 1 (n= 30; 21%) RR 0.89 (p=0.528)<br>Grade 2 (n=20; 32%) RR 1.22 (p=0.349)<br>Grade 3 (n=16; 38%) RR 1.33 (p=0.215)<br>Grade 4 (n=32; 82%) RR 2 (<0.001)<br><br>**Cerebral palsy**<br>Grade 0 (n=54; 7%)<br>Grade 1 (n= 12; 8%) RR 1.16 (p=0.63)<br>Grade 2 (n=10; 13%) RR 1.97 (p=0.037)<br>Grade 3 (n=10; 18%) RR 2.7 (p=0.002)<br>Grade 4 (n=30; 39%) RR 6.07 (p<0.001)<br><br>**Moderate or severe cerebral palsy**<br>Grade 0 (n=20; 2%)<br>Grade 1 (n= 4; 3%) RR 1.04 (p=1)<br>Grade 2 (n=4; 5%) RR 2.13 (p=0.143)<br>Grade 3 (n=4; 7%) RR 2.91 (p=0.062)<br>Grade 4 (n=9; 12%) RR 4.91 (p<0.001)<br><br>**Bilateral blindness**<br>Grade 0 (n=6; 1%)<br>Grade 1 (n= 1; 1%) RR 0.9 (p=1)<br>Grade 2 (n=1; 1%) RR 0.9 (p=0.471)<br>Grade 3 (n=0; 0%) RR 0 (p=1)<br>Grade 4 (n=2; 3%) RR 3.42 (p<0.154)<br><br>**Bilateral deafness**<br>Grade 0 (n=5; 1%)<br>Grade 1 (n=3; 2%) RR 3.25 (p=0.114)<br>Grade 2 (n=2; 3%) RR 4.21 (p=0.117)<br>Grade 3 (n=1; 2%) RR 2.96 (p=0.326)<br>Grade 4 (n=1; 1%) RR 2.05 (p=0.429)<br><br>**Mental development index (mean +/- SD; Difference in means (p value))**<br>No IVH 97.2+/- 18.8<br>Grade 1 97.6 +/- 18.7; 0.5 (p=0.471)<br>Grade 2 92.5 +/- 23.4; -4.6 (p=0.227)<br>Grade 3 89.4 +/- 25.2; -7.7 (p=0.056)

SUPPLEMENTAL FIGURE 15
Continued.

				Grade 4 77.5 +/- 23.4; -19.7 (P<0.001)
33<br>#	Sarkar 2018[49]<br><br>Retrospective cohort study<br><br>USA	**Population**<br>• Gestation 22-27 weeks<br>• Born 2002-2012<br><br>**Exposure**<br>• Persistent cPVL (n=87)<br>• Late cPVL (n=270)<br>• Disappearing cPVL (n=76)<br><br>**Comparator**<br>• Matched<br>• No PVL on imaging (n=6630)<br><br>**Ascertainment/ definition**<br>• NICHD neonatal research network<br>• Ultrasound or MRI used<br>• Based on most severe findings<br>• Persistent (present in first 28 days and at 36 weeks' gestation)<br>• Late (first detected at 36 weeks)<br>• Disappearing (present in first 28 days but not present by 36 weeks' gestation)	**Outcomes**<br>• Neurodevelopmental impairment (composite)<br>• Cognitive<br>• Motor<br>• Cerebral palsy<br>• Speech and language<br>• Visual impairment<br>• Hearing impairment<br><br>**Assessment/ measurement**<br>• BSID II (before 2007)<br>• BSID III (after 2007)<br>• GMFCS<br><br>**Follow-up**<br>• 18-26 months corrected<br>• Follow up >87%	**Neurodevelopmental impairment**<br>Disappearing cPVL, n=33, 52%<br>No cPVL n=1406, 27%<br>OR 3.00 95% CI (1.82, 4.94) p<0.01<br>aOR 1.17 95% CI (1.04,1.31) p=0.01<br><br>Persistent cPVL<br>aOR 1.21 95% CI (1.06,1.37) p<0.01<br><br>Late cPVL<br>aOR 1.16 (1.06,1.27) p<0.01<br><br>**No neurodevelopmental impairment**<br>Disappearing cPVL, n=27, 43%<br>No cPVL n=4004, 76%<br>OR 0.23 95% CI (0.14, 0.38) p<0.01<br><br>**Cognitive**<br>**BSID II MDI <70**<br>Disappearing cPVL, n=10, 71%<br>No cPVL n=746, 39%<br>OR 3.93 (1.23, 12.59) P=0.01<br><br>**BSID III cognitive score <70**<br>Disappearing cPVL, n=16, 33%<br>No cPVL n=316, 10%<br>OR 4.70 95% CI (2.55, 8.67) P<0.01<br><br>**BSID III cognitive score <85**<br>Disappearing cPVL, n=24, 50%<br>No cPVL n=943, 29%<br>OR 2.49 95% CI (1.41, 4.40) P<0.01<br><br>**BSID III cognitive score, mean (SD)**<br>Disappearing cPVL, 47.2 (12.5)<br>No cPVL 51.9 ± 8.0<br><br>**Motor**<br>**BSID II PDI <70**<br>Disappearing cPVL, n=9, 64%<br>No cPVL n=551, 29%<br>OR 4.45 95% CI (1.48, 13.34) p<0.01<br><br>**BSID III motor <70**<br>Disappearing cPVL, n=13, 37%<br>No cPVL n=302, 13%<br>OR 3.98 95% CI (1.98, 7.98) p<0.01<br><br>**BSID III motor <85**<br>Disappearing cPVL, n=21, 60%<br>No cPVL n=772, 33%<br>OR 3.04 95% CI (1.54, 6.01) p<0.01<br><br>**Moderate/ severe CP**<br>Disappearing cPVL, n=22, 35%<br>No cPVL n=355, 7%<br>OR 7.37 95% CI (4.34, 12.51) p<0.01<br><br>**Speech Language**<br>**BSID III language score <70**<br>Disappearing cPVL, n=18, 38%<br>No cPVL n=611, 19%<br>OR 2.57 95% CI (1.43, 4.65) p<0.01<br><br>**BSID III language score <85**<br>Disappearing cPVL, n=28, 58%<br>No cPVL n=1653, 51%<br>OR 1.34 95% CI (0.75, 2.39) p=0.32<br><br>**BSID language score, mean (SD)**<br>Disappearing cPVL,78.9 (21.9)<br>No cPVL 83.6 (16.7)
34	Shankaran 2004[51]<br><br>Prospective cohort<br><br>USA	**Population (n=1046)**<br>• Gestation ≤24 weeks<br>• Birthweight ≤750g<br>• 1-min Agar ≤3<br>• Born 1993-1999<br><br>**Exposure**<br>• IVH 3-4 (n=90)<br>• cPVL (n=25)<br><br>**Comparison**<br>• Unmatched<br>• No specific comparison group selected<br>• Remaining preterm infants<br>• No IVH 3-4 or cPVL.<br><br>**Ascertainment/ definition**<br>• NICHD neonatal research network	**Outcomes**<br>• Cerebral palsy<br>• Cognitive<br>• Motor<br>• Blindness<br>• Hearing impairment<br>• Death<br>• Neurodevelopmental impairment (composite)<br><br>**Assessment/ measurement**<br>• Neurological examination (Amiel-Tison neurologic assessment)<br>• BSID II (MDI, PDI)<br>• Developmental evaluation<br>• Medical and social history<br><br>**Follow-up**<br>• 18-22 months<br>• 78% follow-up of survivors	**Predictors of a mental development index <70**<br>**Grade III-IV IVH**<br>OR 2.5, 95% CI 1.4-4.4;<br>aOR 1.8 (0.9-3.6)<br><br>**Cystic PVL**<br>OR 4.1 (1.4-11.6)<br>aOR 3.4 (1.0-10.8)*<br><br>**Predictors of a psychomotor development index <70**<br>**Grade III-IV IVH**<br>OR 1.9 (1.0-3.3),<br>aOR 1.1 (0.6-2.3)<br><br>**Cystic PVL**<br>OR 3.1 (1.2-7.8)<br>aOR 3.1 (1.1-9.1)<br><br>**Predictors of cerebral palsy**<br>**Grade III-IV IVH**<br>OR 2.4 (1.3-4.5)<br>aOR 1.9 (0.9-4.1)<br><br>**Cystic PVL**<br>OR 5.1 (1.9-13.6)

SUPPLEMENTAL FIGURE 15
Continued.

				aOR 4.4 (1.4-13.5)<br><br>**Any neurodevelopmental impairment**<br>**Grade III-IV IVH**<br>OR 3.1 (1.7-5.7)<br>aOR 2.5 (1.2-5.2)<br><br>**Cystic PVL**<br>OR 4.2 (1.2-14.6)<br>aOR 2.4 (0.6-9.5)<br><br>**Neurodevelopmental impairment or death after NICU discharge**<br>**Grade III-IV IVH**<br>OR 3.1 (1.7-5.7)<br>aOR 2.4 (0.6-9.5)<br><br>**Cystic PVL**<br>OR 4.0 (1.2-14.0)<br>aOR 2.7 (0.7-10.1)
35	Shankaran 2020[50]<br><br>Retrospective cohort<br><br>USA	**Population**<br>• Gestation ≤26 weeks<br>• Born 2011-2015<br><br>**Exposure**<br>• IVH grade 1-2 (n=769)<br>• IVH 3-4 (n=815)<br><br>**Comparison**<br>• Unmatched<br>• Normal cranial ultrasound (n=2632)<br><br>**Ascertainment/ definition**<br>• NICHD neonatal research network<br>• Most severe findings used<br>• Evaluated by clinical radiologist	**Outcomes**<br>• Neurodevelopmental impairment (composite)<br>• Cognitive<br>• Motor<br>• Cerebral palsy<br>• Visual impairment<br>• Hearing impairment<br><br>**Assessment/ measurement**<br>• BSID III<br><br>**Follow-up**<br>• 18-26 months<br>• >92% follow-up	**Neurodevelopmental impairment**<br>IVH 1-2 n=93, 18.4%<br>Comparator n=266, 13.8%<br>aOR 1.34 95% CI (1.01-1.78)<br><br>IVH 3-4 n=211, 46.6%<br>Comparator n=266, 13.8%<br>aOR 4.96 95% CI (3.84-6.41)<br><br>**Normal outcome (no impairment)**<br>IVH 1-2 n=265, 51.4%<br>Comparator n=1105, 56.6%<br>aOR 0.84 95% CI (0.67-1.04)<br><br>IVH 3-4 n=123, 26,7%<br>Comparator n=1105, 56.6%<br>aOR 0.28 95% CI (0.22-0.36)<br><br>**Cognitive**<br>**BSID III cognitive score >85**<br>IVH 1-2 n=283, 54.1%<br>Comparator n=1141, 58%<br>aOR 0.85 95% CI (0.69-1.06)<br><br>IVH 3-4 n=150, 33.2%<br>Comparator n=1141, 58%<br>aOR 0.37 95% CI (0.29-0.47)<br><br>**BSID III cognitive score 70-85**<br>IVH 1-2 n=181, 34.6%<br>Comparator n=672, 34.2%<br>aOR 1.00 95% CI (0.80-1.25)<br><br>IVH 3-4 n=168, 37.2%<br>Comparator n=672, 34.2%<br>aOR 1.05 95%CI (0.82-1.33)<br><br>**BSID III cognitive score 55-69**<br>IVH 1-2 n=58, 11.1%<br>Comparator n=135, 6.9%<br>aOR 1.79 95% CI (1.26-2.53)<br><br>IVH 3-4 n=111, 24.6%<br>Comparator n=135, 6.9%<br>aOR 4.11 95% CI (3.01-5.61)<br><br>**BSID III cognitive score <55**<br>IVH 1-2 n=1, 0.2%<br>Comparator n=18, 0.9%<br><br>IVH 3-4 n=23, 5.1%<br>Comparator n=18, 0.9%<br><br>**Motor**<br>**BSID III motor score >85**<br>IVH 1-2 n=290, 57.1%<br>Comparator n=1168, 60%<br>aOR 0.91 95% CI (0.72-1.14)<br><br>IVH 3-4 n=149, 34.3%<br>Comparator n=1168, 60%<br>aOR 0.35 95% CI (0.27-0.45)<br><br>**BSID III motor score 70-85**<br>IVH 1-2 n=154, 30.3%<br>Comparator n=605, 31.1%<br>aOR 0.97 95% CI (0.76-1.22)<br><br>IVH 3-4 n=125, 28.8%<br>Comparator n=605, 31.1%<br>aOR 0.86 95% CI (0.66-1.11)<br><br>**BSID III motor score 55-69**<br>IVH 1-2 n=41, 8.1%<br>Comparator n=110, 5.7%<br>aOR 1.37 95% CI (0.91-2.05)<br><br>IVH 3-4 n=77, 17.7%<br>Comparator n=110, 5.7%<br>aOR 3.39 95% CI (2.40-4.79)

SUPPLEMENTAL FIGURE 15
Continued.

				**BSID III motor score <55**<br>IVH 1-2 n=23, 4.5%<br>Comparator n=63, 3.2%<br>aOR 1.25 95% CI (0.74-2.11)<br><br>IVH 3-4 n=83, 19.1%<br>Comparator n=63, 3.2%<br>aOR 5.77 95 CI (3.90-8.52)<br><br>**Cerebral palsy**<br>IVH 1-2 n=68, 12.6%<br>Comparator n=178, 8.8%<br>aOR 1.47 95% CI (1.06-2.04)<br><br>IVH 3-4 n=202, 42.9%<br>Comparator n=178, 8.8%<br>aOR 7.16 95% CI (5.46-9.38)<br><br>**Hearing impairment**<br>IVH 1-2 n=11, 2.1%<br>Comparator n=48, 2.4%<br>aOR 0.87 95% CI (0.43-1.76)<br><br>IVH 3-4 n=25, 5.3%<br>Comparator n=48, 2.4%<br>aOR 1.87 95% CI (1.08-3.23)<br><br>**Visual impairment**<br>IVH 1-2 n=4, 0.7%<br>Comparator n=12, 0.6%<br>aOR 0.92 95% CI (0.25-3.34)<br><br>IVH 3-4 n=27, 5.8%<br>Comparator n=12, 0.6%<br>aOR 9.04 95%CI (4.28-19.29)
36	Tu 2019[52]<br><br>Retrospective cohort study<br><br>Taiwan	**Population**<br>• Gestation < 32 weeks<br>• Birthweight <1500g<br>• Admitted to NICU 2003-2012<br><br>**Exposure**<br>• cPVL n=33<br>• IVH 1-2 n=142<br>• IVH 3-4 n=34<br>• IVH 3-4 and. cPVL (n=7)<br><br>**Comparator**<br>• Unmatched<br>• No significant brain injury (n=626)<br><br>**Ascertainment/ definition**<br>• Ultrasounds performed by pediatric radiologists or neonatologists	**Outcomes**<br>• Cognitive<br>• Cerebral palsy<br>• Epilepsy<br><br>**Assessment/ measurement**<br>• Wechsler Preschool and Primary Scale of Intelligence–Revised (5 years)<br>• Gross motor function score<br>• Parental interview<br><br>**Follow-up**<br>• 6 months, 12 months, 24 months (5 years)<br>• 85% follow-up of survivors	**Epilepsy**<br>IVH 1-2 n=2, 10.5%<br>IVH 3-4 N=7. 21%<br>Comparator n=6, 1%<br>OR 13.6 (4.9-35.7) p<0.001<br>aOR 7.9 95% CI (2.2, 28.3) p<0.01<br><br>cPVL n=9, 27%<br>Comparator n=6, 1%<br>OR 15.2 (5.4-45.5) p<0.001<br>aOR = 13.5 95%CI (4.1, 44.4) p<0.001<br><br>**Drug resistant epilepsy**<br>IVH 3-4 n=6, 75%<br>OR 30.0 (2.2-406) P<0.01<br><br>cPVL n=6,75% p=0.65<br><br>**Cognitive, full scale IQ mean (SD)**<br>**IVH 3-4 and epilepsy**<br>63.8 (4.2)<br>**IVH 3-4 without epilepsy**<br>73.8 (19.0) P<0.001<br><br>**Comparator with epilepsy**<br>67.5 (16.3)<br>**Comparator without epilepsy**<br>88.9 (13.6) p<0.001<br><br>**Cerebral palsy**<br>**IVH 3-4 and epilepsy**<br>N=11, 85%<br>**IVH 3-4 without epilepsy**<br>N=20, 43% p=0.02<br><br>**Comparator with epilepsy**<br>n=0, 0%<br>**Comparator without epilepsy**<br>n=21, 3% P=1
37	Wang 2017[53]<br><br>Prospective cohort<br><br>Taiwan	**Population**<br>• Gestation <31 weeks<br>• Birthweight <1500g<br>• Born 2001-2012<br><br>**Exposure**<br>• Isolated cPVL (n=93)<br>• cPVL with IVH grade 1-2 (n=118)<br>• cPVL with IVH grade 3 (n=75)<br><br>**Comparison**<br>• Unmatched<br>• No IVH or cPVL (n=4633)<br><br>**Ascertainment/ definition**<br>• Imaging performed and interpreted by pediatric neurologists<br>• DeVries classification of IVH	**Outcomes**<br>• Developmental delay<br>• Cerebral palsy<br>• Death<br><br>**Measurement/ assessment**<br>• BSID II (MDI)<br>• Algorithm-based classification of cerebral palsy based on gross motor function<br>**Follow-up**<br>• 24 months<br>• 88.3% follow-up of survivors	**Isolated cPVL**<br>Cerebral palsy (n=53; 63.1%)<br>Developmental delay (n=35; 41.7%)<br><br>**cPVL with IVH 1-2**<br>Cerebral palsy (n=79; 73.8%)<br>Developmental delay (n=55; 51.4%)<br><br>**cPVL with IVH 3-4**<br>Cerebral palsy (n=61; 88.4%)<br>Developmental delay (n=43; 62.3%)<br><br>**Comparison group**<br>Cerebral palsy (n=120; 3.2%)<br>Developmental delay (n=201; 5.3%)<br><br>**Death (<28 days)**<br>n=1503 (15%)
38	Wy 2015[54]<br><br>Retrospective cohort<br><br>USA	**Population**<br>• Gestation <37 weeks<br>• Birthweight <2500g<br>• Born 1985-1988	**Outcomes**<br>• Cognitive<br>• Behavioral<br>• Speech and language	**Cognitive**<br>Stanford Binet IQ (n); mean(SE)<br>IVH grade 1-2 (n=93); 85.4 (2.3)<br>No IVH (n=270); 88 (1.8)<br><br>Difference in mean IQ scores (95%CI; p-value)

SUPPLEMENTAL FIGURE 15
Continued.

		**Exposure**<br>• IVH grade 1-2 (n=93)<br><br>**Comparison**<br>• Unmatched<br>• No IVH (n=273)<br><br>**Ascertainment/ definition**<br>• Data from Infant Health and Development Program (a national multisite randomized control trial of early educational intervention)	**Measurement/ assessment**<br>• Stanford–Binet Intelligence scales<br>• Wechsler Intelligence Scale for Children<br>• Wechsler Abbreviated Scale of Intelligence<br>• The Peabody Picture Vocabulary Test-Revised<br>• Woodcock–Johnson Tests of Achievement<br>• Achenbach Behavior Checklist<br>• Child Behavior Checklist<br><br>**Follow-up**<br>• 3 years (8 years and 18 years)<br>• 71% follow-up at 18 years	2.59 (-1.51, 6.69; p=0.21)<br><br>**Behavior**<br>Achenbach Total Prob. Sum Score (n); mean (SE)<br>IVH grade 1-2 (n=88); 49.4 (2.8)<br>No IVH (n=263); 49.1 (2.1)<br><br>Difference in mean scores (95%CI; p-value)<br>-0.34 (-5.27, 4.59; p=0.89)<br><br>**Speech and language**<br>Peabody Picture Vocabulary Test – Revised (n); mean (SE)<br>IVH grade 1-2 (n=75); 82.6 (2.2)<br>No IVH (n=240); 85.7 (1.6)<br><br>Difference in mean scores (95% CI; p-value)<br>3.14 (-0.73, 7; p=0.11

SUPPLEMENTAL FIGURE 15

Continued.

	Selection (*satisfactory; No = not satisfactorily done; n/a)				Comparability (*satisfactory; No = not satisfactorily done; n/a)		Exposure or Outcome (*satisfactory; No = not satisfactorily done; n/a)			Subtotal assessment				Additional comments
	1	2	3	4	1a	1b	1	2	3	Selection (0-1 = Poor; 2 = Fair; 3+ Good)	Comparability (0 = poor; 1 = fair; 2+ = good)	Exposure or Outcome (0 = poor; 1 = fair; 2+ = good)	Total score: 0-3 high risk of bias; 4-6 moderate risk of bias 7-9 low risk of bias	
Adams-Chapman 2018[17]	*	*	*	No (deafness or blindness could have been congenital)	*	*	*	*	*	Good	Good	Good	8	They present p values for the logistic regression but no odds ratios or confidence intervals. Unable to access population characteristics for the exposure and comparator groups of interest e.g. IVH 3-4 merged with cPVL.
Adant 2019[18]	No	*	*	* (excluded those with congenital abnormalities)	*	*	No	*	No	Good	Good	Fair	6	Population not representative - focus of study was spontaneous intestinal perforation. Infants without IVH didn't have brain injury excluded per se (but did not have IVH 3-4 on ultrasound). Independent outcome assessment but not blinded; telephone survey of parents. High numbers lost to follow-up. Table 3 contains errors with respect to outcomes (MDI and PDI mislabeled as motor and cognitive respectively).
Altendahl 2021[19]	*	*	*	* (given the types of outcomes assessed)	No	*	*	*	No	Good	Fair	Good	7	Study focuses on comparing outcomes of those with ROP compared to those without ROP. There's a subgroup analysis of outcomes after IVH which we have been able to include but for this population the exposed and comparator children are not matched. The multivariable mode adjusts for sex, birthweight, IVH grade, public insurance and age at testing.

SUPPLEMENTAL FIGURE 16

Quality scores of studies exploring outcomes after intracranial hemorrhage or preterm white matter injury. BPD, bronchopulmonary dysplasia; NEC, necrotising enterocolitis; PDA, patent ductus arteriosus; ROP, retinopathy of prematurity; SGA, small for gestational age.

Ancel 2006[20]	*	*	*	* (CP couldn't be present at birth)	*	No	*	*	*	Good	Fair	Good	8	No apparent adjustment for factors other than gestation and no description of baseline characteristics for brain-injured vs nonbrain-injured participants<br><85% follow-up for enrolled infants; but does give clear description of those lost to follow-up and no significant differences with respect to US brain injury findings between groups<br>Half of infants born at 32 weeks' gestation in 2 regions excluded 'randomly' to ease follow-up workload but no description of how randomization was done
Bae 2018[21]	*	*	*	* (Excluded those with congenital abnormalities)	*	*	*	*	No	Good	Good	Good	8	Matched on gestational age and birthweight. Similar baseline characteristics between exposure and comparator groups. Pediatric radiologist undertook all scans. Excluded infants with congenital anomalies and IVH.<br>Low numbers.<br>No adjustment for confounders.
Bae 2021[22]	*	*	*	* (Excluded those with congenital abnormalities)	*	*	*	*	No	Good	Good	Good	8	Excluded infants with major congenital abnormalities or major brain injury (high grade IVH 3-4 or PVL).<br><br>Significant differences between IVH and no IVH in terms of gestation and birthweight.<br><br>Adjusted for RDS, treated PDA, BPD, severe ROP, gestation, SGA, NEC, sepsis.

SUPPLEMENTAL FIGURE 16
Continued.

Banihani 2019[23]	*	*	*	* (given the types of outcomes assessed)	*	*	*	*	*	Good	Good	Good	9	Matched on sex, gestational age and month of birth.<br><br>Similar baseline characteristics (except preterm rupture of membranes and out-born location of birth)<br><br>The comparison group still includes infants with brain injury (IVH. 1-3; PVL. meningitis and ventriculomegaly).
Benavente-Fernandez 2019[24]	*	*	*	* (given the types of outcomes assessed)	*	*	*	*	No	Good	Good	Good	8	Excluded infants with congenital malformations or syndrome, congenital infections or large hemorrhage infarctions (>2 cm) on ultrasound.<br>No matching.<br><br>From 2011, only preterm infants who received magnesium sulphate were included.<br><br>Odds ratios adjusted for gestation, small for gestational age, chronic lung disease.<br><br>Only 84.2% had maternal level of education available. Used multiple imputation for missing data. Those lost to follow-up were more likely to have missing data.

SUPPLEMENTAL FIGURE 16
Continued.

Bolisetty 2019[26]	*	*	*	* (excluded those with congenital abnormalities)	*	*	*	*	No	Good	Good	Good	8	Excluded those with major congenital malformations.<br><br>Adjusted for gestation, sex, birthweight <10th percentile, late onset sepsis, chronic lung disease and postnatal steroids for chronic lung disease.<br>Multivariate analysis exploring IVH 3-4 as a risk factor is comparing those with IVH 3-4 to those with IVH 0-2. Unable to extract denominator data for those without IVH.
Bolisetty 2014[25]	*	*	*	* (excluded those with major congenital malformations, eg,deafness and blindness)	*	*	*	*	*	Good	Good	Good	9	Excluded those with major congenital malformation. <85% follow-up but clear description of those lost given; similar proportions of IVH between groups.<br><br>Overall inter-rater reliability diagnosing IVH was good 78-90% but poor for IVH 1 (45%), IVH 2 (41%), and IVH 3 (38%).
Broitman 2007[27]	*	*	*	No (deafness or blindness could have been congenital)	*	*	*	*	*	Good	Good	Good	8	<85% follow-up but clear description of those lost given; similar proportions of IVH between groups but PVL rate different - small number and magnitude of difference probably would not significantly influence overall results

SUPPLEMENTAL FIGURE 16
Continued.

Chen 2004[28]	No	*	*	* (given the types of outcomes assessed)	No	No	*	*	*	Good	Poor	Good	6	Retrospective study - only infants with complete developmental follow-up at 6, 12, and 18 months included, thus representativeness of cohort not clear Exposed and non-exposed cohorts not matched for gestation or birthweight; Babies in persistent periventricular echogenicity group less mature and lower birthweight with *p* value approaching significance
Chmait 2019[29]	No	*	*	No (visual or hearing impairment could be congenital)	No	No	*	*	*	Fair	Poor	Good	5	Unmatched comparators and no adjustment for confounders. Very select population (survivors of twin-to-twin transfusion syndrome).
DaSilva 2018[31]	*	*	*	No (could have had congenital deafness)	*	No	*	*	*	Good	Fair	Good	7	Appears significant difference between IVH and no IVH groups regarding weight and sex, though papers states this did not have a confounding effect. Exposed and comparator groups reportedly similar in terms of gestation and risk of hearing loss but no matching or adjustment specified
DeMauro 2020[32]	*	*	*	* (given the types of outcomes assessed)	*	*	*	*	No	Good	Good	Good	8	Brain injury excluded from comparator group.<br><br>Adjusted for gestation, sex, small for gestational age (SGA), antenatal steroids, 5-minute Apgar ≤5, race,<br>BPD, PDA, sepsis, NEC requiring surgery, severe ROP, postnatal steroids, and corrected age at assessment, including center as a random effect.

SUPPLEMENTAL FIGURE 16
Continued.

Duncan 2019[33]	*	*	*	* (given the types of outcomes assessed)	*	*	*	*	*	Good	Good	Good	9	Adjusted for center, birth weight, gestation, multiple gestation, race and ethnicity, Medicaid enrolment, sex, antenatal steroids, cesarean delivery, late-onset sepsis, surgery for ROP, NEC, PDA, postnatal steroids, and BPD.
Haslam 2018[34]	*	*	*	* (excluded those with congenital abnormalities)	*	*	*	*	*	Good	Good	Good	9	Adjusted for ethnicity, parental employment status, antenatal steroids, maternal substance use, gestation, sex, score for neonatal acute physiology II, late onset sepsis, and BPD.<br><br>Unclear if comparison group in logistic regression for IVH grade 3-4 includes all other infants (including those with IVH grade 1-2) or those without IVH.
Hintz 2015[35]	no	*	*	No (could have had congenital deafness)	*	*	*	*	*	Fair	Good	Good	7	Authors state cohort was a selective subgroup of babies enrolled in a larger randomized trial comparing surfactant with CPAP. No clear description of inclusion criteria for the NEURO subcohort.
Klebermass -Schrehof 2012[36]	*	*	*	No (could have had congenital blindness)	*	*	*	*	No	Good	Good	Good	7	No clear description of number lost to follow-up, though mentions that follow-up rate at 5.5 years was 54-61%
Kratimenos 2019[37]	*	*	*	* (given the types of outcomes assessed)	No	*	*	*	No	Good	Fair	Good	7	Excluded those with incomplete medical records, major congenital malformations, congenital anemia, or chromosomal abnormality.<br><br>No matching or stratification.<br>Adjusted for significant covariates such as 1-minute Apgar score.

SUPPLEMENTAL FIGURE 16
Continued.

Lean 2019[38]	*	*	*	* (given the types of outcomes assessed)	*	*	*	*	*	Good	Good	Good	9	Excluded those with chromosomal abnormalities, congenital infections, in-utero drug exposure or poor cord gases.<br><br>Brain injury excluded from comparator group.<br><br>Similar gestation for brain injured and comparator preterm group.<br><br>Adjusted for intrauterine growth restriction, oxygen therapy at 36 weeks, no maternal antenatal steroids, postnatal steroids, NEC, PDA, ROP, culture positive sepsis, MRI length and height measurements, and length of parental nutrition.
Lin 2020[39]	*	*	*	* (excluded those with congenital abnormalities)	*	*	*	*	No	Good	Good	Good	8	Excluded infants with chromosomal anomaly (or genetic disorders) or major congenital malformations.<br><br>Comparator group likely includes those with other brain injuries e.g. IVH grade 1-2. Adjusted for gender, maternal age, paternal age, gestation, antenatal steroids, mode of delivery, respiratory distress syndrome, acidosis at birth, IVH 3-4 and cPVL.
Logan 2011[40]	*	*	*	* (given the types of outcomes assessed)	*	*	*	*	*	Good	Good	Good	9	Amount of overlap between exposure types unclear from data presented.<br>Study was designed to assess the association between hypotension and outcomes rather than the association between white matter damage and outcomes.
Miller 2005[42]	*	*	*	* (given the types of outcomes assessed)	*	*	*	*	*	Good	Good	Good	9	Unclear if other abnormalities present in comparator group.
Patra 2006[44]	*	*	*	* (excluded those with major congenital	*	*	*	*	*	Good	Good	Good	9	Multivariate logistic regression adjusted for the socio-demographic and neonatal risk factors. Maternal marital status, race, and

SUPPLEMENTAL FIGURE 16
Continued.

				malformations, eg, deafness and blindness)										education as well as neonatal gender, chronic lung disease, sepsis, and NEC were included as covariates.
Pavaine 2016[45]	*	*	*	* (excluded those with congenital or chromosomal abnormalities)	*	*	*	*	No	Good	Good	Good	8	Follow-up rate below 85% and no description of those lost to follow up.
Payne 2013[46]	*	*	*	* (excluded those with congenital anomalies)	*	*	*	*	*	Good	Good	Good	9	Adjusted for: IVH severity, gestation, sex, race and ethnicity, maternal educational level, chorioamnionitis, sepsis, antenatal steroids, postnatal steroid use, high-frequency ventilation, and PDA.
Peixoto 2018[47]	*	*	*	* (excluded those with congenital malformations)	*	No	*	*	No	Good	Fair	Good	7	Excluded infants with congenital malformations, genetic syndromes, cPVL, cerebellar hemorrhage, or focal infarction. Matched on gestation and year of birth.
Radic 2015[48]	*	*	*	No (deafness or blindness could have been congenital)	*	*	*	*	*	Good	Good	Good	8	Unmatched comparator group and no adjustment for covariates. The covariates associated with IVH and disability are presented but not adjusted for.
Sarkar 2018[49]	*	*	*	No (visual or hearing impairment could be congenital)	*	*	*	*	*	Good	Good	Good	8	Unclear if other brain injuries, eg, IVH excluded from comparator group. Adjusted for gestation, antenatal steroids, chorioamnionitis, sex, race, maternal education, bilateral presence of blood or echodensity in the ventricles or parenchyma (using screening cranial ultrasonography results within the first 28 days), late-onset sepsis, medical or surgical NEC, and BPD.
Shankaran 2004[51]	no	*	*	No (deafness or blindness could have been congenital)	*	*	*	*	*	Fair	Good	Good	7	Study selected infants with birthweight <750g and 1-minute Apgar <3 - thus findings regarding association of brain injury with neurodevelopmental outcomes are not generalizable. Note <85% follow-up but appears no major differences in group lost to follow-up.

SUPPLEMENTAL FIGURE 16

Continued.

Shankaran 2020[50]	*	*	*	* (excluded infants with major congenital anomalies)	*	*	*	*	*	Good	Good	Good	9	Excluded infants who died within 12 hours, with major anomaly, who did not receive intensive care following birth and with missing cranial ultrasound or hemorrhage status.<br><br>Adjusted for center, sex, antenatal steroids, chorioamnionitis, hypertension, mode of delivery, mother's education, and gestational age.
Tu 2019[52]	*	*	*	* (given the types of outcomes assessed)	*	*	*	No	*	Good	Good	Good	8	Comparator group of infants who did not have significant brain injury included those with milder brain injury, eg, IVH 1-2. Participants were followed-up at different ages (6 months to 5 years) and some of these time-points would have been too soon to detect certain outcomes, eg, cerebral palsy.<br>Adjusted for gestation, sex, NEC, neonatal seizure, IVH 3-4 and cPVL.
Wang 2017[53]	*	*	*	* (given the types of outcomes assessed)	*	*	*	*	*	Good	Good	Good	9	Significant differences in gestation and birthweight between exposed and nonexposed cohorts appear to have been adjusted for in analysis. Follow-up rate for cPVL group 89%.

SUPPLEMENTAL FIGURE 16
Continued.

Wy 2015[54]	No	No	No	* (given the types of outcomes assessed)	*	*	*	*	(*)	Poor	Good	Good	6	Follow-up 71%. Paper states no differences in demographics, or in outcomes at 3 and 8 years of those who failed to attend at 18 years. Only a minority of the main cohort underwent cranial ultrasound and authors state no standards or criteria for performance of ultrasound – it was at the physician's discretion. Those without imaging were not included and those who were imaged represent a biased sample.
**Case-control studies**														
Choi 2020[30]	*	*	No	*	*	*	*	*	*	Fair	Good	Good	8	Low numbers of those with brain injury. Lack of information on those without brain injury and no detail about systematic effort to rule out brain injury in comparators, eg, through imaging.

SUPPLEMENTAL FIGURE 16
Continued.

Matsushita 2019[41]	No	*	No but appropriate	*	*	*	*	*	No	Fair	Good	Good	6	Excluded infants with major congenital abnormalities, chromosomal aberrancy, central nervous system disorders (anencephaly, meningocele, fetal hydrocephaly and holoprosencephaly).<br><br>Unclear if brain injury, eg, IVH grade 1-2 excluded from comparison group in logistic regression.<br><br>Adjusted for gestation, birthweight, maternal age, multiple births, chorioamnionitis, antenatal steroids, caesarean section, sex, Apgar, small for gestational age, respiratory distress syndrome, moderate to severe bronchopulmonary dysplasia, sepsis, patent ductus arteriosus, IVH grade 3-4 (in cPVL), cPVL (in IVH grade 3-4), necrotizing enterocolitis, retinopathy of prematurity, cerebral palsy, and developmental quotient <70.
Nair 2021[43]	*	*	No but appropriate	*	*	*	*	*	No	Good	Good	Good	7	Those with incomplete records excluded. Adjusted for receipt of furosemide, patent ductus arteriosus ligation, severe retinopathy of prematurity, bronchopulmonary dysplasia, home oxygen on discharge. Comparator infants likely included those with other brain injuries.

SUPPLEMENTAL FIGURE 16
Continued.

Study or Subgroup	IVH grade 1-2 Events	IVH grade 1-2 Total	No IVH Events	No IVH Total	Weight, %	Odds Ratio M-H, Random, 95% CI
**Bayley Scale of Infant Development II**						
Bolisetty 2014	55	296	126	1041	33.1	1.66 (1.17-2.34)
Broitman 2007	176	395	515	1308	41.3	1.24 (0.99-1.55)
Patra 2006	49	104	72	257	25.6	2.29 (1.43-3.67)
**Subtotal (95% CI)**		**795**		**2606**	**100.0**	**1.60 (1.14-2.24)**
Total events	280		713			
Heterogeneity: Tau² = 0.06; Chi² = 6.04, df = 2 (*P* = .05); I² = 67						
Test for overall effect: Z = 2.70 (*P* = .007)						
**Bayley Scales of Infant and Toddler Development III**						
Bae 2021	6	45	19	195	3.7	1.43 (0.53-3.80)
Payne 2013	81	270	276	1021	40.5	1.16 (0.86-1.55)
Shankaran 2020	93	769	266	2632	55.8	1.22 (0.95-1.57)
**Subtotal (95% CI)**		**1084**		**3848**	**100.0**	**1.20 (1.00-1.45)**
Total events	180		561			
Heterogeneity: Tau² = 0.00; Chi² = 0.20, df = 2 (*P* = .90); I² = 0%						
Test for overall effect: Z = 1.93 (*P* = .05)						

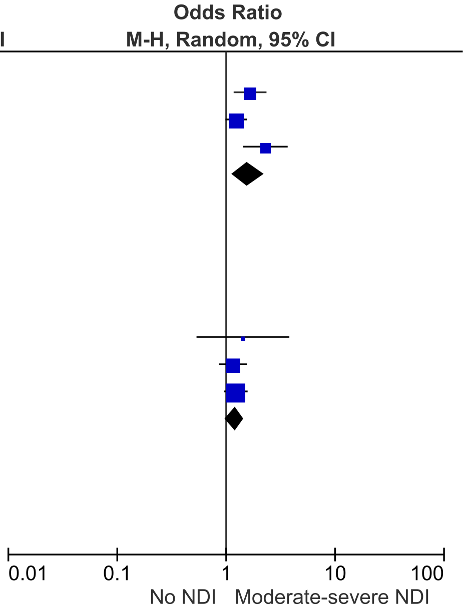

Test for subgroup differences: Chi² = 2.04, df = 1 (*P* = .15), I² = 51.0%

SUPPLEMENTAL FIGURE 17

Sensitivity analysis of the impact of outcome assessment tools on risk of neurodevelopmental impairment after IVH grade 1-2.

Study or Subgroup	IVH grade 1-2 Events	IVH grade 1-2 Total	No IVH Events	No IVH Total	Weight, %	Odds Ratio M-H, Random, 95% CI
Broitman 2007	78	395	246	1308	37.4	1.06 (0.80-1.41)
Klebermass-Schrehof 2012	27	113	39	303	30.3	2.13 (1.23-3.68)
Patra 2006	47	104	65	260	32.3	2.47 (1.53-3.99)
**Total (95% CI)**		**612**		**1871**	**100.0**	**1.72 (0.96-3.10)**
Total events	152		350			

Heterogeneity: Tau² = 0.22; Chi² = 11.29, df = 2 (*P* = .004); I² = 82%
Test for overall effect: Z = 1.81 (*P* = .07)

Odds Ratio
M-H, Random, 95% CI

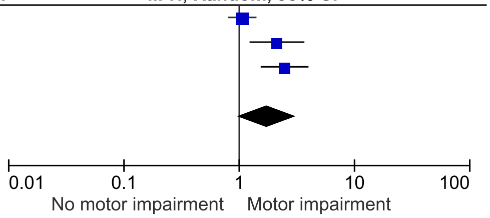

SUPPLEMENTAL FIGURE 18
Forest plot of the combined crude risk of a BSID II Psychomotor Development Index (PDI) score <70 after IVH grade 1-2.

Study or Subgroup	IVH grade 1-2 Events	IVH grade 1-2 Total	No IVH Events	No IVH Total	Weight, %	Odds Ratio M-H, Random, 95% CI
**Children born before 2000**						
Ancel 2006	40	397	100	1469	23.0	1.53 (1.04-2.25)
Broitman 2007	68	395	132	1308	24.5	1.85 (1.35-2.54)
Klebermass-Schrehof 2012	49	132	34	281	20.3	4.29 (2.59-7.09)
Patra 2006	8	100	9	300	11.3	2.81 (1.05-7.50)
Payne 2013	24	267	82	1025	20.9	1.14 (0.71-1.83)
**Subtotal (95% CI)**		**1291**		**4383**	**100.0**	**1.99 (1.30-3.05)**
Total events	189		357			
Heterogeneity: Tau² = 0.17; Chi² = 16.35, df = 4 (*P* = .003); I² = 76%						
Test for overall effect: Z = 3.16 (*P* = .002)						
**Children born after 2000**						
Bae 2021	0	45	1	195	0.5	1.42 (0.06-35.55)
Bolisetty 2014	35	336	68	1043	26.4	1.67 (1.09-2.56)
Peixoto 2018	1	86	0	86	0.5	3.04 (0.12-75.55)
Radic 2015	22	227	54	771	17.9	1.42 (0.85-2.40)
Shankaran 2020	68	540	178	2016	54.7	1.49 (1.11-2.00)
**Subtotal (95% CI)**		**1234**		**4111**	**100.0**	**1.53 (1.22-1.90)**
Total events	126		301			
Heterogeneity: Tau² = 0.00; Chi² = 0.44, df = 4 (*P* = .98); I² = 0%						
Test for overall effect: Z = 3.77 (*P* = .0002)						

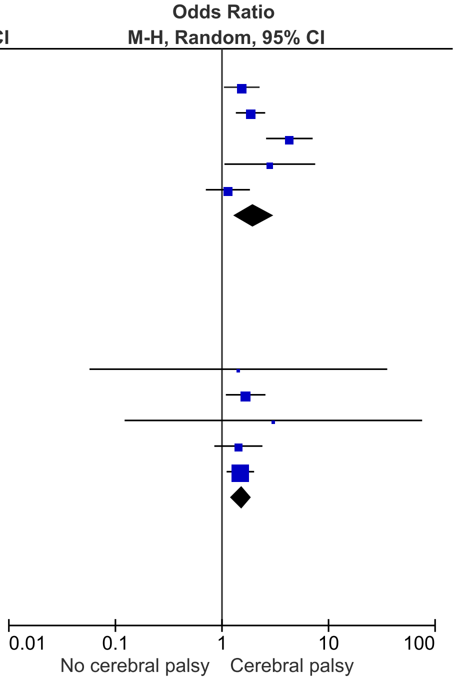

Test for subgroup differences: Chi² = 1.18, df = 1 (*P* = .28), I² = 15.1%

SUPPLEMENTAL FIGURE 19

Sensitivity analysis of the risk of cerebral palsy for infants with IVH grade 1-2 born before and after 2000.

Study or Subgroup	IVH grade 1-2 Events	IVH grade 1-2 Total	No IVH Events	No IVH Total	Weight, %	Odds Ratio M-H, Random, 95% CI
Bolisetty 2014	15	263	31	900	21.4	1.70 (0.90-3.19)
Broitman 2007	133	395	417	1308	30.3	1.08 (0.85-1.38)
Klebermass-Schrehof 2012	28	113	36	305	23.3	2.46 (1.42-4.27)
Patra 2006	47	104	63	258	25.0	2.55 (1.58-4.12)
**Total (95% CI)**		**875**		**2771**	**100.0**	**1.79 (1.09-2.95)**
Total events	223		547			

Heterogeneity: Tau² = 0.20; Chi² = 14.77, df = 3 (*P* = .002); I² = 80%
Test for overall effect: Z = 2.28 (*P* = .02)

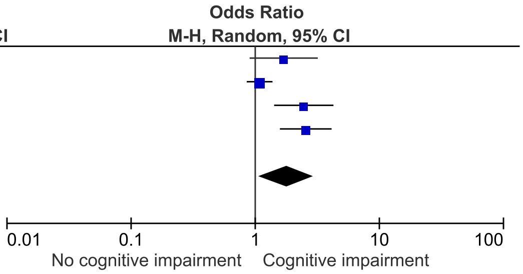

SUPPLEMENTAL FIGURE 20
Forest plot of the crude risk of BSID II MDI <70 in infants with IVH grade 1-2.

Study or Subgroup	log[Odds Ratio]	SE	Weight, %	Odds Ratio IV, Random, 95% CI
Broitman 2007	1.23837423	0.12750161	58.9	3.45 (2.69-4.43)
Shankaran 2004	0.64185389	0.28166764	41.1	1.90 (1.09-3.30)
**Total (95% CI)**			**100.0**	**2.70 (1.52-4.80)**

Heterogeneity: $Tau^2 = 0.13$; $Chi^2 = 3.72$, df = 1 (P = .05); $I^2 = 73\%$
Test for overall effect: Z = 3.38 (P = .0007)

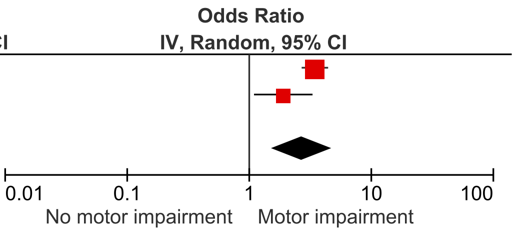

SUPPLEMENTAL FIGURE 21
A forest plot of the crude risk of an abnormal BSID II PDI score (<70) after IVH grade 3-4.

Study or Subgroup	log[Odds Ratio]	SE	Weight, %	Odds Ratio IV, Random, 95% CI
**Children born before 2000**				
Ancel 2006	1.9235	0.2867	30.5	6.84 (3.90-12.01)
Broitman 2007	1.7584	0.1414	41.1	5.80 (4.40-7.66)
Shankaran 2004	0.8755	0.3168	28.4	2.40 (1.29-4.47)
**Subtotal (95% CI)**			**100.0**	**4.75 (2.79-8.10)**
Heterogeneity: Tau² = 0.16; Chi² = 7.46, df = 2 (*P* < .02); I² = 73%				
Test for overall effect: Z = 5.72 (*P* = .00001)				
**Children born after 2000**				
Bolisetty 2014	1.7901	0.2731	9.9	5.99 (3.51-10.23)
Payne 2013	1.4914	0.2011	18.2	4.44 (3.00-6.59)
Radic 2015	1.7424	0.2357	13.2	5.71 (3.60-9.06)
Shankaran 2020	1.5129	0.1119	58.7	4.54 (3.65-5.65)
**Subtotal (95% CI)**			**100.0**	**4.79 (4.05-5.67)**
Heterogeneity: Tau² = 0.00; Chi² = 1.60, df = 3 (*P* = .66); I² = 0%				
Test for overall effect: Z = 18.27 (*P* = .00001)				

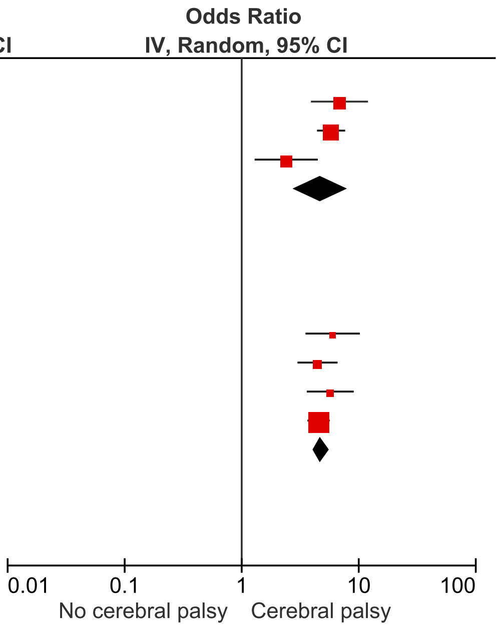

Test for subgroup differences: Chi² = 0.00, df = 1 (*P* = .98), I² = 0%

SUPPLEMENTAL FIGURE 22

Sensitivity analysis of the risk of cerebral palsy for infants with IVH grade 3-4 born before and after 2000.

Study or Subgroup	log[Odds Ratio]	SE	Weight, %	Odds Ratio IV, Random, 95% CI
Bolisetty 2014	1.7917595	0.3722192	27.0	6.00 (2.89-12.44)
Broitman 2007	0.64006675	0.1216053	41.3	1.90 (1.49-2.41)
Shankaran 2004	0.91629073	0.2921256	31.7	2.50 (1.41-4.43)
**Total (95% CI)**			**100.0**	**2.83 (1.54-5.20)**

Heterogeneity: Tau² = 0.22; Chi² = 8.93, df = 2 (*P* = .01); I² = 78%
Test for overall effect: Z = 3.34 (*P* = .0008)

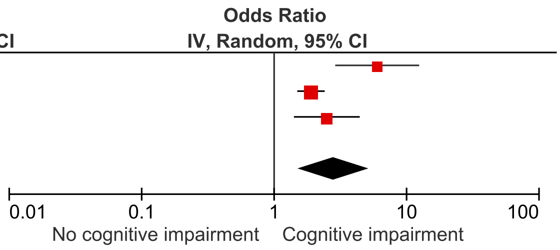

SUPPLEMENTAL FIGURE 23

Forest plot of the crude risk of ‘abnormal’ motor scores on BSID II (<70) after IVH grade 3-4.

Study or Subgroup	IVH grade 3-4 Events	IVH grade 3-4 Total	No IVH Events	No IVH Total	Weight, %	Odds Ratio M-H, Random, 95% CI
Banihani 2019	5	20	8	40	6.1	1.33 (0.37-4.77)
Payne 2013	80	181	255	1021	93.9	2.38 (1.72-3.30)
**Total (95% CI)**		**201**		**1061**	**100.0**	**2.30 (1.67-3.15)**
Total events	85		263			

Heterogeneity: Tau² = 0.00; Chi² = 0.74, df = 1 (*P* = .39); I² = 0%

Test for overall effect: Z = 5.16 (*P* = .00001)

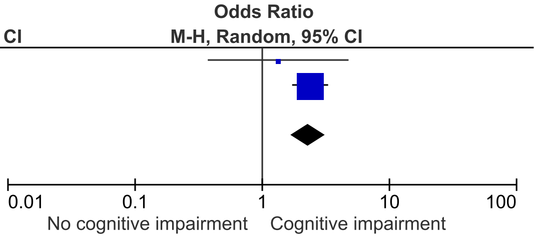

SUPPLEMENTAL FIGURE 24

Forest plot of the crude risk of 'abnormal' motor scores on BSID III (<85) after IVH grade 3-4.

Study or Subgroup	log[Odds Ratio]	SE	Weight, %	Odds Ratio IV, Random, 95% CI
Broitman 2007	1.58320859	0.33637148	33.1	4.87 (2.52-9.42)
Sarkar 2018 (disappearing cPVL only)	1.09861229	0.25472674	57.7	3.00 (1.82-4.94)
Shankaran 2004	1.43508453	0.63742346	9.2	4.20 (1.20-14.65)
**Total (95% CI)**			**100.0**	**3.63 (2.49-5.31)**

Heterogeneity: Tau² = 0.00; Chi² = 1.38, df = 2 (*P* = .50); I² = 0%

Test for overall effect: Z = 6.67 (*P* = .00001)

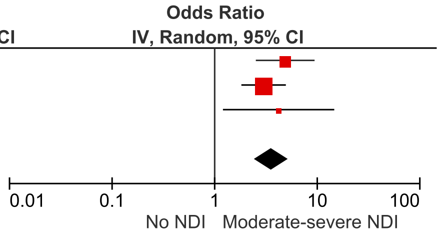

SUPPLEMENTAL FIGURE 25

Forest plot of the crude risk of moderate to severe neurodevelopmental impairment (NDI) after cPVL.

Study or Subgroup	log[Odds Ratio]	SE	Weight, %	Odds Ratio IV, Random, 95% CI	Odds Ratio IV, Random, 95% CI
Lin 2019	1.62531126	0.25912029	36.1	5.08 (3.06-8.44)	
Sarkar 2018 (late cPVL)	0.14842001	0.04610918	38.6	1.16 (1.06-1.27)	
Shankaran 2004	0.87546874	0.70462179	25.2	2.40 (0.60-19.55)	
**Total (95% CI)**			**100.0**	**2.38 (0.73-7.70)**	

Heterogeneity: Tau² = 0.93; Chi² = 32.42, df = 2 (*P* = .00001); I² = 94%

Test for overall effect: Z = 1.44 (*P* = .15)

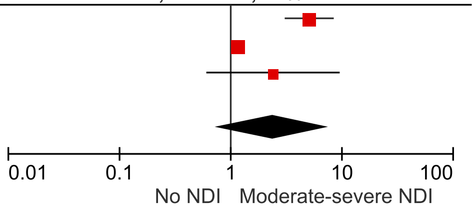

SUPPLEMENTAL FIGURE 26

Forest plot of the adjusted risk of moderate to severe nerodevelopmental impairment (NDI) after cPVL.

Study or Subgroup	log[Odds Ratio]	SE	Weight, %	Odds Ratio IV, Random, 95% CI
Broitman 2007	2.03731662	0.30288918	50.6	7.67 (4.24-13.89)
Sarkar 2018	1.4929041	0.56089922	21.6	4.45 (1.48-13.36)
Shankaran 2004	1.13140211	0.47750056	27.8	3.10 (1.22-7.90)
**Total (95% CI)**			**100.0**	**5.30 (3.00-9.36)**

Heterogeneity: Tau² = 0.07; Chi² = 2.78, df = 2 (*P* = .00001); I² = 28%
Test for overall effect: Z = 5.75 (*P* = .15)

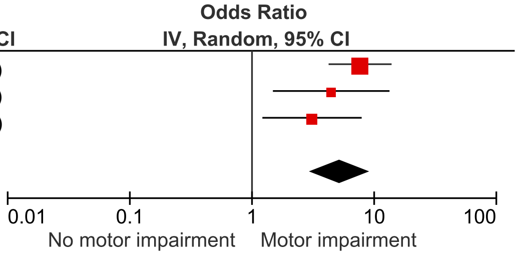

SUPPLEMENTAL FIGURE 27
Forest plot of the crude risk of motor impairment (BSID II PDI <70) after cPVL.

Study or Subgroup	log[Odds Ratio]	SE	Weight, %	Odds Ratio IV, Random, 95% CI
Ancel 2006	3.1420166	0.2555198	21.1	23.15 (14.03-38.20)
Broitman 2007	2.76243635	0.30860625	20.2	15.84 (8.65-29.00)
Sarkar 2018	1.99741771	0.27006479	20.9	7.37 (4.34-12.51)
Shankaran 2004	1.62924054	0.50209589	16.3	5.10 (1.91-13.64)
Wang 2017	3.70594652	0.22908856	21.6	40.69 (25.97-63.75)
**Total (95% CI)**			**100.0**	**14.91 (7.30-30.46)**

Heterogeneity: Tau² = 0.56; Chi² = 31.05, df = 4 (*P* = .00001); I² = 87%
Test for overall effect: Z = 7.41 (*P* = .15)

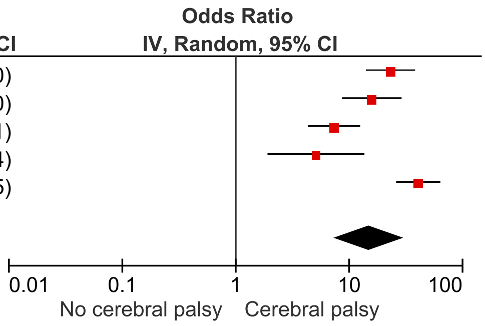

SUPPLEMENTAL FIGURE 28
Forest plot of the crude risk of cerebral palsy after cPVL.

Study or Subgroup	log[Odds Ratio]	SE	Weight, %	Odds Ratio IV, Random, 95% CI
Broitman 2007	1.1662709	0.2944981	64.7	3.21 (1.08-5.72)
Sarkar 2018	1.3686394	0.5933389	16.0	3.93 (1.23-12.57)
Shankaran 2004	1.410987	0.5394216	19.3	4.10 (1.42-11.80)
**Total (95% CI)**			**100.0**	**3.48 (2.18-5.53)**

Heterogeneity: $Tau^2 = 0.00$; $Chi^2 = 0.21$, $df = 2$ ($P = .00001$); $I^2 = 0\%$
Test for overall effect: $Z = 5.26$ ($P = .15$)

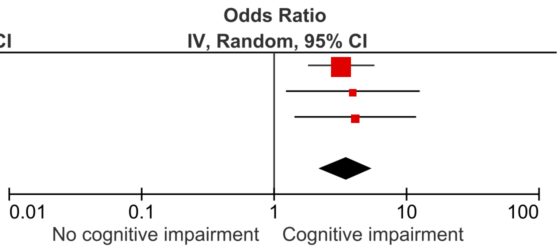

SUPPLEMENTAL FIGURE 29
Forest plot of the crude risk of cognitive impairment (BSID II MDI score <70) after WMI.
